# Ovarian dysfunction following prenatal exposure to an insecticide, chlordecone, associates with altered epigenetic features

**DOI:** 10.1186/s13072-019-0276-7

**Published:** 2019-05-13

**Authors:** Louis Legoff, Ouzna Dali, Shereen Cynthia D’Cruz, Antonio Suglia, Aurore Gely-Pernot, Chloé Hémery, Pierre-Yves Kernanec, Abbassia Demmouche, Christine Kervarrec, Sergei Tevosian, Luc Multigner, Fatima Smagulova

**Affiliations:** 1 EHESP, Inserm, Irset (Institut de recherche en santé, environnement et travail) - UMR_S 1085, Univ Rennes, 35000 Rennes, France; 2 Biotoxicology Laboratory, Department of Biology, Faculty of Natural Sciences and Life, Djillali Liabes University, 22000 Sidi Bel Abbès, Algeria; 3 Department of Physiological Sciences, University of Florida, Box 100144, 1333 Center Drive, Gainesville, FL 32610 USA

## Abstract

Chlordecone (CD) is an insecticide that was used in the French West Indies for several years to control the banana root borer pest. Given its nonsignificant degradation, it persists in the environment. CD is a carcinogenic compound with reproductive and developmental toxicity and is a recognized endocrine-disrupting chemical. In this study, we examined the effects of CD on female reproductive system of mice with the focus on epigenetic features in ovary. Our data show that gestational exposure to low dose of CD affects meiotic double-strand breaks repair in female embryos. In adult mice derived from CD-treated pregnant females, we observed delayed puberty, decreased number of primordial and increased number of atretic follicles. Gene expression analysis revealed that *Rcbtb2* and *Rbpms* genes were not expressed in embryonic gonads. Estrogen signaling- and oocyte maturation-associated genes were downregulated in adult ovaries. The morphological changes were associated with altered epigenetic features: increased H2Aub and increased H3K27me3 and decreased H4ac and H3K4me3 in embryonic oocytes. The DNA damage-associated, γH2AX marks were detected in the follicles of treated but not control adult ovaries. We also found reduced H3K4me3 and H4ac in fully grown oocytes of the treated ovaries. The ChIP-seq analysis of H3K4me3 in adult ovaries showed that target genes of ZFP57 and TRIM28, which regulate pluripotency and imprinting, were significantly enriched in altered regions. Our study clearly demonstrates that gestational exposure to a low dose of CD impairs the function of female reproductive system and the changes are associated with altered epigenetic features.

**Electronic supplementary material:**

The online version of this article (10.1186/s13072-019-0276-7) contains supplementary material, which is available to authorized users.

## Introduction

Environmental factors are known to affect numerous biological processes, particularly development, due to high proliferative activity of cells during organogenesis. While the ability of environmental compounds to cause changes in the DNA makeup of the developing cell is well known, the ability of the chemicals to alter epigenetic processes in the cell is just beginning to be understood. During mammalian development, two rounds of epigenetic reprogramming occur to promote distinct cellular fates that would give rise to germ cells. The first event occurs in preimplantation embryo and the second one in the primordial germ cells when the cells undergo the somatic-to-germline transition (SGT) (e.g., reviewed in [1]). During SGT, germ cells of both sexes reset their epigenetic marks and become committed to oocyte or male gonocyte fates. Consequently, the developmental window of SGT is very sensitive to exogenous environmental factors.

In male animals, environmentally induced changes have been shown to induce stable modifications in the mammalian germline and contribute to transgenerational effects [2–5]. In some cases, toxicant-induced effects were detected up to the fourth generation of males in the absence of the initial stimulus [6]. In contrast to male gonocytes, the embryonic ovarian germ cells after SGT undergo meiosis, a specialized division process that gives rise to haploid gametes. Subsequently, meiosis gets arrested in females just before birth. During meiosis, DNA is particularly vulnerable to environmental exposure because meiosis entails the formation of double-strand DNA breaks (DSBs) throughout the entire genome. DSBs formation is an integral part of the homologous recombination process, with the major goal of bringing together the parental chromosomes before first meiotic division. DSBs formation normally occurs at discrete regions of the genome known as recombination hotspots; up to 25000 hotspots were found in the mouse genome in meiotic cells [7]. Hence, exposure to toxicants at this stage is likely to result in chromosome missegregation and the formation of abnormal gametes.

Meiosis in females remains dormant until puberty and is completed only after fertilization. Successful meiosis requires the orchestrated actions of a comprehensive histone modification ensemble, which include histone phosphorylation, trimethylation, acetylation, ubiquitination, among others [8, 9]. Histone H2A and H2B ubiquitination are critical for meiosis. For instance, H2A ubiquitination (H2Aub) in meiotic prophase cells was observed at the heterochromatic X and Y chromosomes in males [10], as well as at unsynapsed chromosomes of both sexes [11]. Ubiquitination of H2A is also important for DNA repair; these marks are abundant at DNA damage sites [12]. It is suggested that E3 ubiquitin ligase RNF8 catalyzes the ubiquitination of H2A and H2AX at damaged sites [13, 14]. On the other hand, H2A ubiquitination is also critical for polycomb repressor complex 1 (PRC1)-dependent repression of developmental genes, which is required to maintain embryonic stem (ES) cell identity [15]. During this process, polycomb repressor complex 2 (PRC2) is first recruited to the developmental genes where it catalyzes the trimethylation of histone H3 at lysine 27 (H3K27me3) [16, 17]. Genes with H3K27me3 marks are subsequently targeted by PRC1, and their chromatin acquires ubiquitination at histone H2A. This PRC1/PRC2 combination regulates, for example, Hox gene family and targets of *Oct4/Nanog/Sox2* genes. Monoubiquitination of H2A at developmental genes is catalyzed by E3 ligases, such as RING1A and RING1B [18]. Thus, histone H2Aub appears to play an essential role in gene regulation and maintain DNA integrity during meiosis.

Recent studies have shown that growing oocytes in arrested meiosis undergo another specialized reprogramming event [19]. Specifically, the fully grown oocytes acquire the non-canonical broad domains of histones H3K4me3 [19, 20]. Notably, DNA methylation in growing oocytes negatively correlates with these broad peaks, which suggests that blocking DNA methylation at these domains is essential for the early histone-mediated developmental program after fertilization [19]. Both the establishment of the broad H3K4me3 domains in the oocyte and their timely removal past fertilization are critical for zygote genome activation (ZGA) and early embryogenesis [19, 20]. Some of the broad peaks, however, are preserved in late embryos which points to their potential role in later development [20].

During early mammalian development, reversal of histone modifications plays an equally important role as their addition. Many cellular factors including nuclear receptors are implicated in the regulation of histone demethylation and thereby could influence the preservation/removal of histone marks. For example, estrogen receptors (ERs) regulate the activity of histone demethylase [21, 22]. ERs, particularly estrogen receptor beta (ESR2), are expressed in many developing tissues, indicating multiple important functions of estrogen signaling during development (reviewed in [23]). Experimental evidence shows that exposure to environmental toxicants such as bisphenol A (BPA) affects developmental processes, at least partially, through binding to estrogen receptors [24]. BPA also modulates meiosis by affecting chromosomal synapsing, DSBs repair or changing the expression of meiotic genes that rely on the ER function. The organochlorine insecticide, chlordecone (CD, also known as Kepone), has also been shown to bind both ESR1 and ESR2 [25, 26]. Our previous studies on the effects of CD on the male reproductive system showed that CD causes transgenerational effects [2]. We showed that transgenerational effects are associated with alterations in histone H3K4me3 methylation marks residing in DNA locations that are strongly enriched for ESR1 binding motifs in directly exposed F1 and non-exposed F3 generation males [2]. Our study demonstrated that histone epigenetic marks could mediate the transmission of CD-promoted effects to subsequent generations via male germline. Since CD is a very stable compound in the environment, it was predicted that it would persist in the French West Indies for many centuries due to its intensive use from 1973 to 1993 to control the banana root borer pest in the region; therefore, the population there is constantly exposed to this toxicant [27]. Hence, it is very important to reveal the mechanistic effects of CD on organ development including the reproductive system to identify new biomarkers of exposure.

In this new study, we examined the effects of CD on murine female reproductive system exposed in utero, with the focus on epigenetic histones marks that are important for oocyte development and maturation. Our results show that gestational exposure to low dose of CD leads to oxidative stress and affects meiosis and gene expression in embryonic ovaries. In the adult animals from the same lineage, we observed delayed puberty and decreased number of primordial (but increased the number of atretic) follicles. The morphological changes promoted by CD are associated with epigenetic changes in both embryonic and adult ovaries. In summary, CD causes deleterious effects on the female reproductive system, and these effects are associated with modified epigenetic features.

## Results

### The increase in 8-oxo-G in embryonic oocytes after CD exposure

**Fig. 1:**
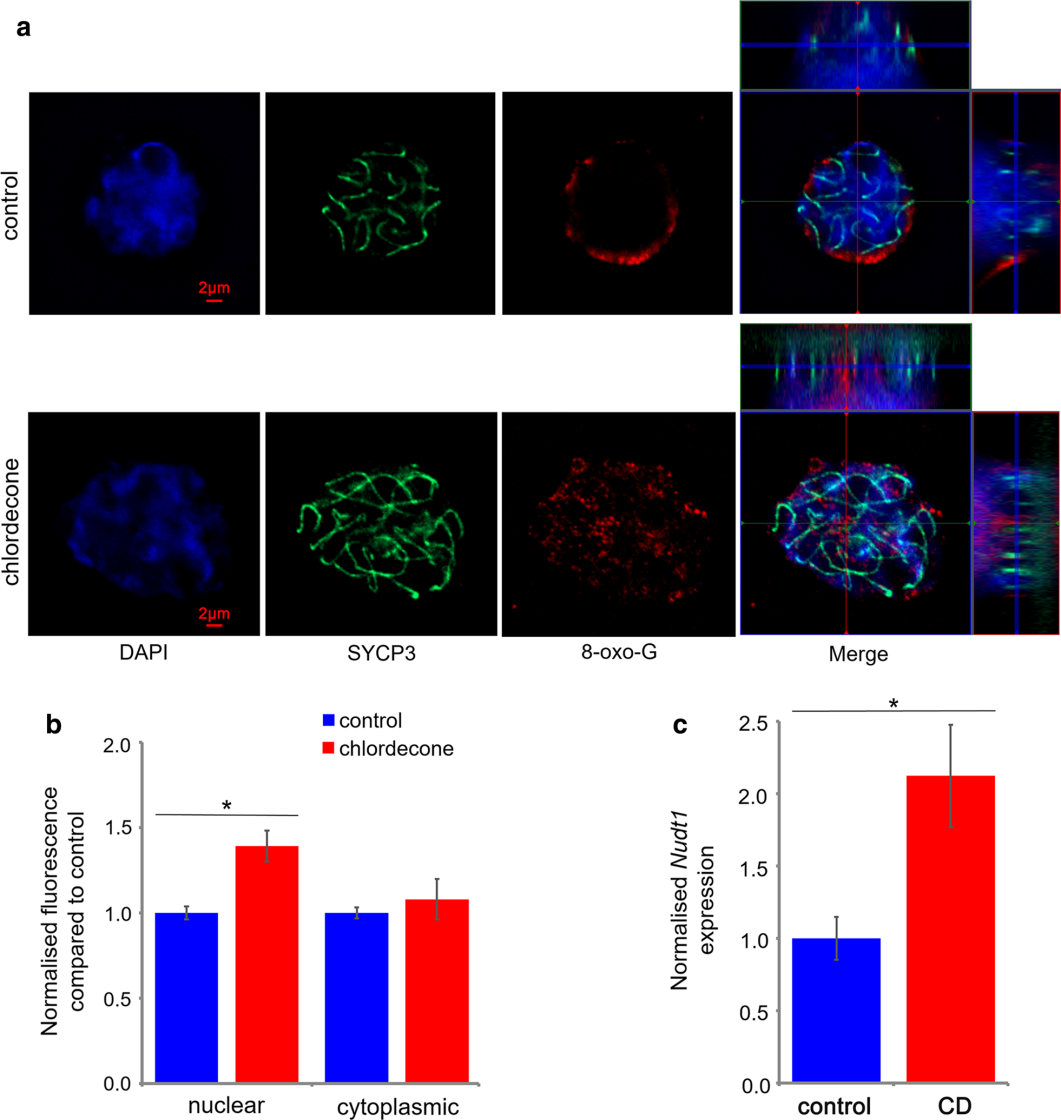
The gestational exposure to CD leads to oxidative stress. Gestational exposure to CD increases the accumulation of 8-oxo-G in fetal oocytes. **a** Representative images of E15.5 oocytes immunostained with antibodies against 8-oxo-G (red), or SYCP3 (green) in structurally preserved nuclei are shown (63X magnification). Given the nuclear 3D structure, we used z-stack image processing technique as described in Methods section, followed by image deconvolution. In the merged image that represents the top view (X/Y axis) of the image, the section views of the X/Z axes (top) and Y/Z axes (right) are also shown. **b** Quantitative analysis of 8-oxo-G staining in E15.5 samples which represents the mean-averaged intensities of 21 individual image planes that were calculated separately in cytoplasm and nucleus. The data were expressed as normalized fluorescence compared to control ± SEM, *p < 0.05, **p < 0.01, ***p < 0.001, *t* test. **c** The normalized mRNA expression level of *Nudt1*, *p < 0.05, *t* test. The gene expression was normalized to the expression of germ cell marker, *Dazl* gene

To reveal whether exposure to CD leads to oxidative stress, we analyzed the levels of 8-oxo-G, which is one of the most common lesions produced in DNA. We performed immunohistochemical analysis of 8-oxo-G by staining the structurally preserved nuclei obtained from E15.5 embryonic ovaries against 8-oxo-G and SYCP3 (a component of the synaptonemal complex to visualize chromosomes) (Fig. 1a) as described in Methods section. Since cells have a spherical form and to better analyze the nuclear content of 8-oxo-G, we performed z-stack and deconvoluted the images (details in Method section). We performed the quantitative analysis of mean-averaged intensities of individual images separately in cytoplasm and nucleus. In E15.5 in CD-exposed oocytes, we found 1.4 times increase in 8-oxo-G signal in the nucleus, but no significant changes were observed in the cytoplasm (Fig. 1b). We determined the mRNA expression of the gene encoding for the enzyme nucleoside diphosphate-linked moiety X-type motif 1 (NUDT1) that eliminates 8-oxo-G and observed 2.1 times increase in its expression (Fig. 1c). These data suggest that exposure to a low dose of CD promotes oxidative stress in embryonic oocytes.

### Meiotic defects are increased in CD-exposed ovaries

**Fig. 2:**
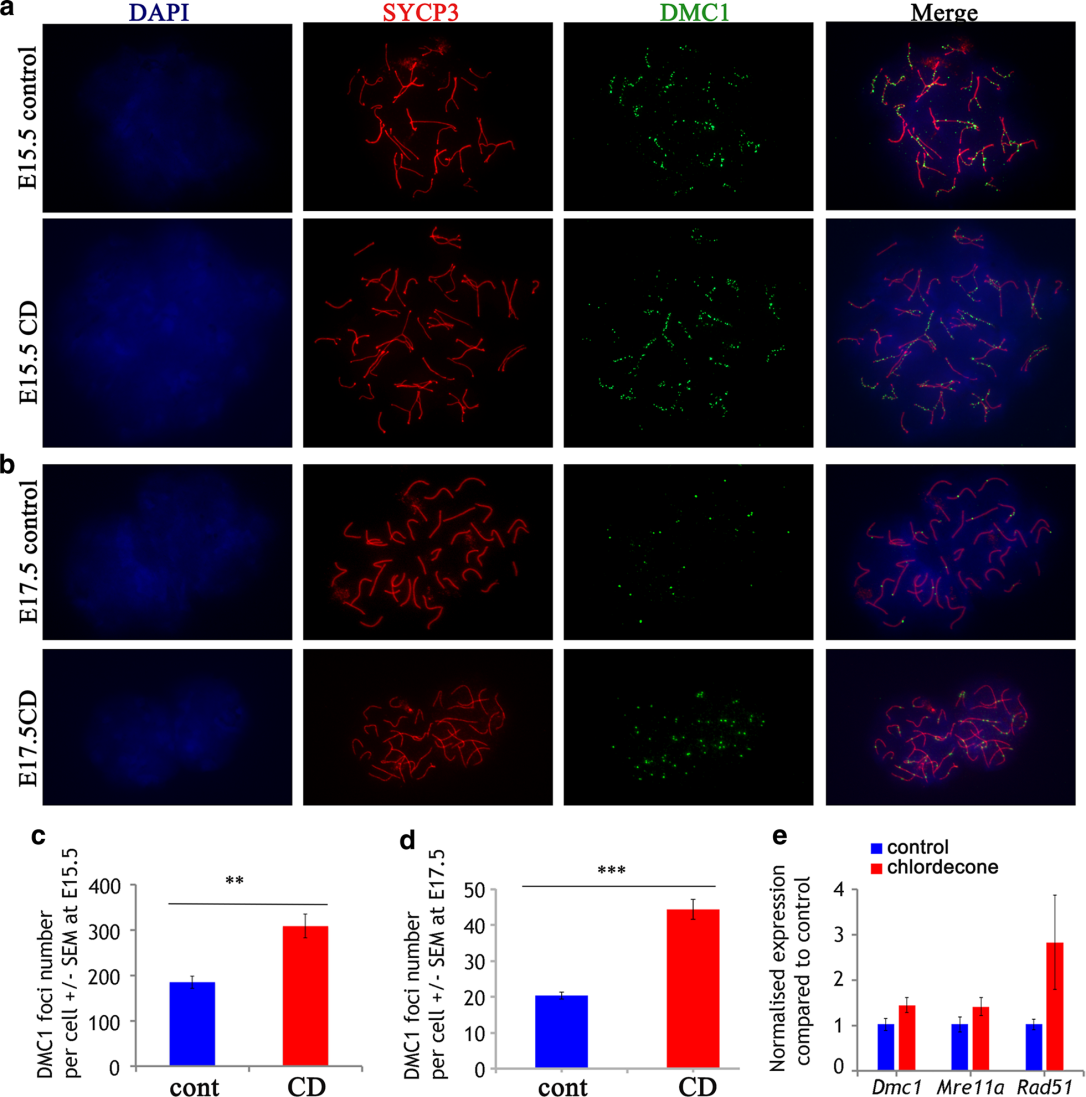
Critical meiotic step is perturbed in embryonic ovaries exposed to CD. **a** Surface spreads from E15.5 control (top row) and CD-treated ovaries (bottom row) were immunostained with anti-DMC1 (green) and anti-SYCP3 (red) antibodies (63X magnification). **b** Surface spreads from E17.5 control and CD-treated ovaries. **c**, **d** Quantitative analysis of the number of DMC1 foci per cell performed on at least twenty of E.15 and 17.5 oocytes from four replicates from different litters, *n* = 4 for each group, **p < 0.01, *t* test. **e** The expression levels of genes involved in DSBs repair were analyzed by RT-qPCR using RNA from E15.5 ovaries from control and CD-treated animals, *p < 0.05, *t* test

To analyze the effects of CD on prophase I of meiosis, we prepared surface spreads from E15.5 ovaries as described in Methods section. We immunostained the spreads for the DNA-binding recombination protein DMC1, and for SYCP3, to visualize the chromosomes (Fig. 2a). DSBs formation and repair is a very dynamic process; the new DSBs appear and get repaired simultaneously, so the numbers of DMC1-stained foci vary from cell to cell. We also analyzed the dynamics of DNA repair by analyzing the DMC1 foci number at E17.5. Most of the breaks are repaired at this time point (Fig. 2b). To evaluate the efficiency of meiotic DNA repair, we calculated the average number of DMC1 foci per oocyte in control and treated groups. The numbers of DMC1 foci at E15.5 were significantly higher in CD-treated oocytes (308 ± 26, CD and 185 ± 14, control), suggesting that CD exposure affects the DNA repair process (Fig. 2c). At E17.5, we found that DSB foci in treated samples (44 ± 3) are still higher compared to control (20 ± 1) (Fig. 2d), which suggests that in exposed ovaries DNA repair process was compromised.

To determine whether ovarian DNA repair is associated with altered activity of DNA repair proteins, we analyzed the RNA expression of the genes that encode major DNA repair proteins (DMC1, RAD51, MRE11A). The mRNA expressions of *Rad51, Mre11a* and *Dmc1* have increased, but the changes were not significant (Fig. 2e). In summary, DSB repair efficiency was altered in CD-exposed oocytes.

### Increase in H2Aub and H3K27me3 and a decrease in H3K4me3 and H4 acetylation after CD exposure

**Fig. 3:**
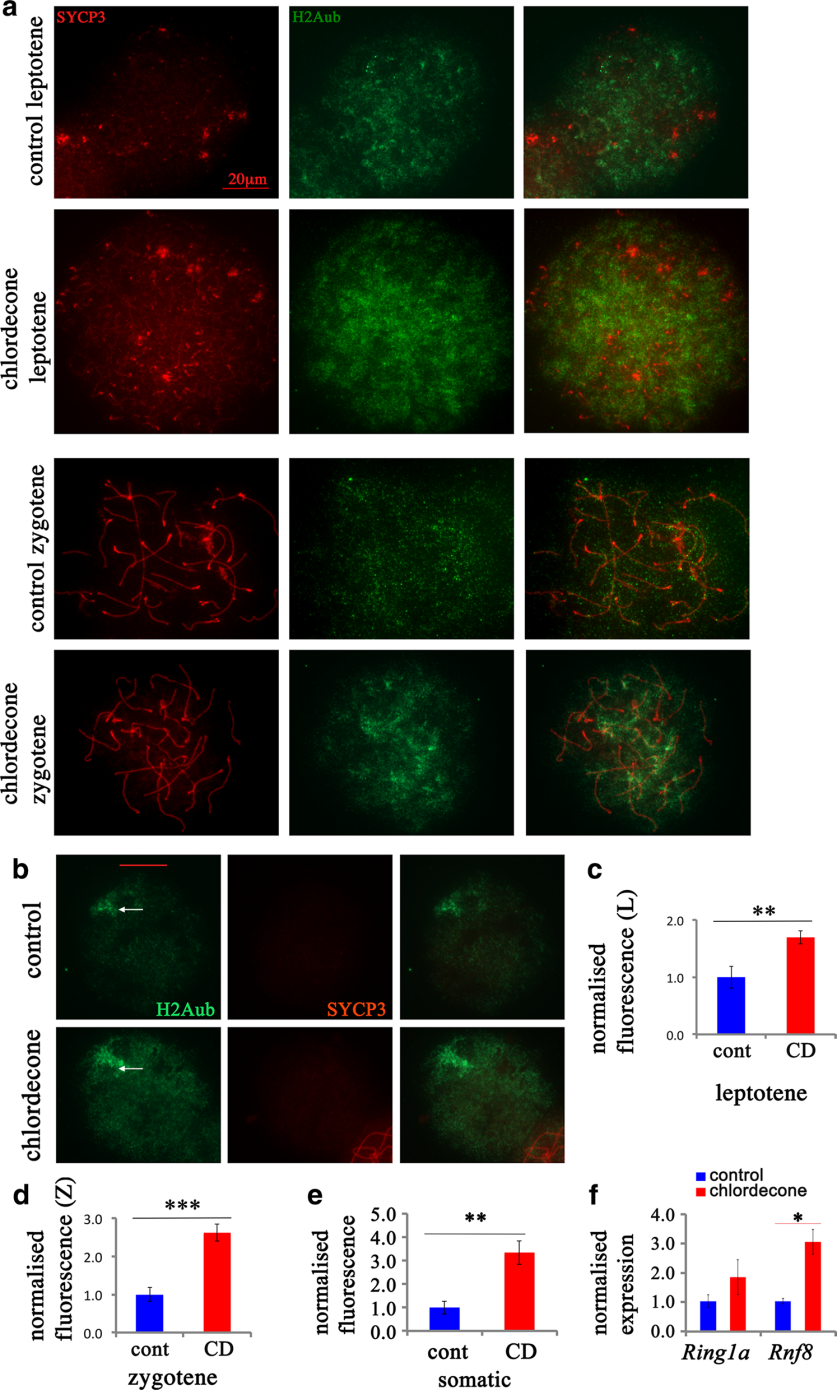
Meiosis defects are associated with altered H2Aub occupancy. **a** Surface spreads from E15.5 ovaries in *leptotene* stage from control (first row) and CD-exposed mice (second row) and in *zygotene* stage from control (third row) and CD-exposed (fourth row) ovaries (63X magnification). Spreads from ovaries were immunostained with anti-H2Aub (green) and anti-SYCP3 (red) antibodies. Quantitative analysis of anti-H2Aub intensity in cells in *leptotene* and (**c**) in *zygotene* (**d**) stages. **b** Representative images of H2Aub in somatic cells. The Barr body is shown with an arrow. Images from 4 control and 5 treatment biological replicates were used for analysis, and the normalized fluorescence intensities in somatic cells (**e**) are provided. The immunofluorescence was performed as described in Methods section using antibodies against H2Aub and SYCP3; the images were obtained using microscope using the fixed exposure time. The images were analyzed using ImageJ software and normalized fluorescence was calculated and the averaged value ± SEM were compared, **p < 0.01, *t* test, the bar represents 20 µm. The analysis was performed on at least 20 oocytes from four replicates for each group, *n* = 4 for each group, *p < 0.05, **p < 0.01, ***p < 0.001, *t* test. **f** The gene expression level of *Rnf8* and *Ring1a* genes was analyzed by RT-qPCR using RNA from E15.5 ovaries from control and CD-treated animals, **p < 0.01, *t* test

**Fig. 4:**
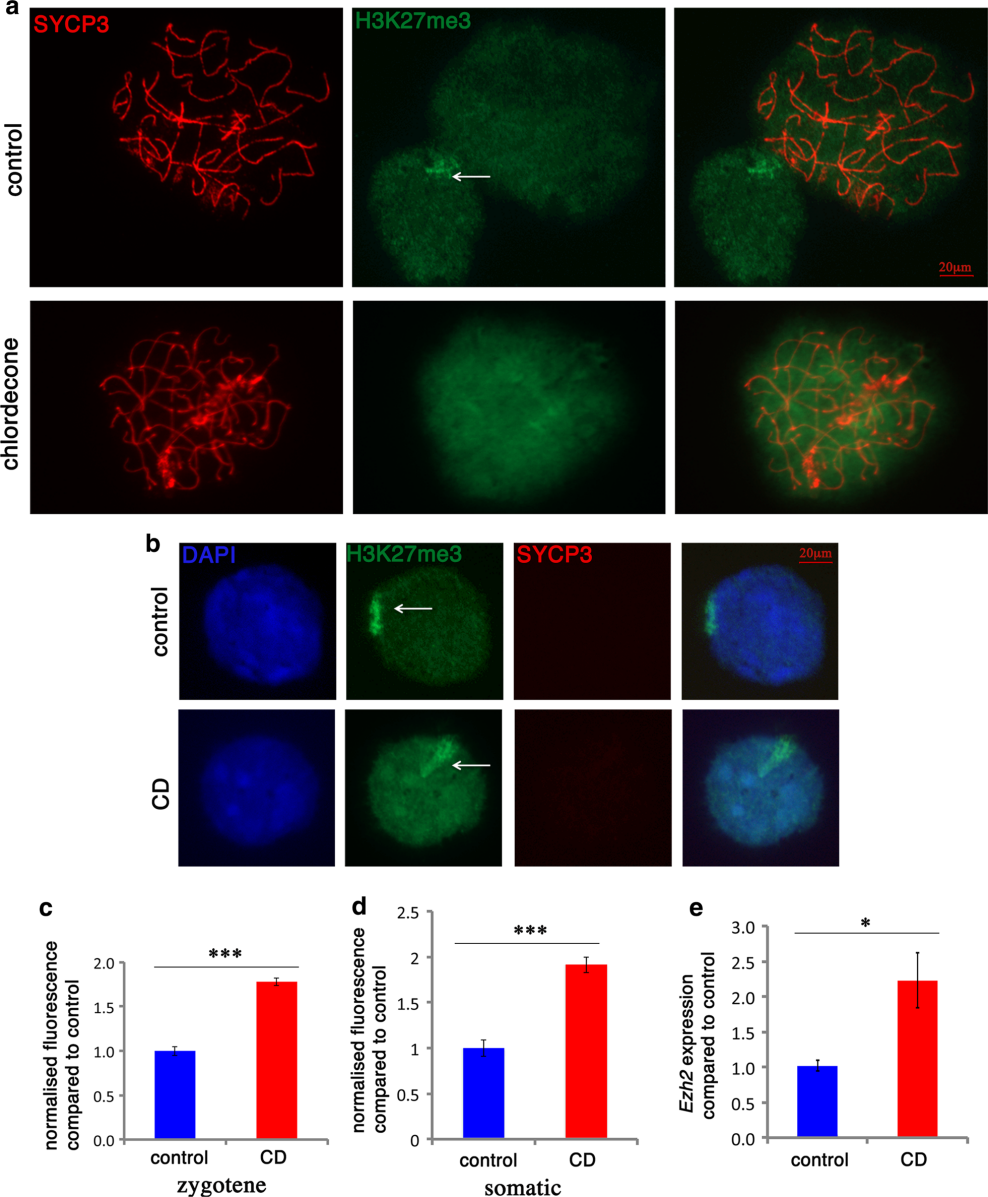
Meiosis defects are associated with altered H3K27me3 occupancy. **a** Surface spreads from E15.5 control (top row) and CD-exposed (bottom row) ovaries were immunostained with anti-H3K27me3 (green) and anti-SYCP3 (red) antibodies, **b** expression of H3K27me3 in somatic cells (63X magnification). The Barr body is shown with an arrow. Quantitative analysis of the anti-H3K27me3 intensity in **c** meiotic cells and **d** somatic cells. The analysis was performed on at least 20 oocytes from four different animals for each group, *n* = 4 for each group, *p < 0.05, **p < 0.01, ***p < 0.001, *t* test. **e** The gene expression level of *Ezh2* was analyzed by RT-qPCR using RNA from E15.5 ovaries from control and CD-treated animals, *p < 0.05, *t* test

To reveal whether gestational exposure to CD affects H2Aub, which is also associated with damaged and unsynapsed chromosomes in the meiotic cells, we immunostained the surface spreads for H2Aub and SYCP3 in E15.5 oocytes. H2Aub in females normally appears as dots around the chromosomes, and it decorates all the chromosomes intensively at the *leptotene* stage, with the signal being decreased at the *zygotene* stage (Fig. 3a and Additional file 1: Fig. S1). In somatic cells (which do not express SYCP3), the signal is mostly associated with the Barr body (Fig. 3b). We performed the quantitative analysis of H2Aub in the cells at the *leptotene* and *zygotene* stage and in somatic cells. In treated samples at *leptotene* stage, the intensity of H2Aub increased 1.7 times (Fig. 3c), at *zygotene* stage the intensity of H2Aub increased 2.6 times (Fig. 3d), and in somatic cells, 3.3 times (Fig. 3e). Since these marks are introduced at damaged sites by the E3 ubiquitin ligase enzyme RNF8 and at pluripotency genes by RING1A, we analyzed their gene expression levels by RT-qPCR. We determined that RNA expression of *Rnf8* increased 3.0 times in exposed ovaries (Fig. 3f); in contrast, *Ring1a* expression was not significantly affected, which suggest that genotoxic effects of CD could explain the increase in H2Aub. Because there is a functional link between the enzymes, which introduce H2Aub and H3K27me3 marks [28, 29], we analyzed the H3K27me3 mark occupancy using immunostaining of surface spreads for H3K27me3 and SYCP3 in E15.5 oocytes. H3K27me3 marks appeared around all chromosomes in meiotic cells (Fig. 4a, and Additional file 1: Fig. S2), with the signal in somatic cells being strong at Barr bodies (Fig. 4b). Quantitative analysis of H3K27me3 intensities showed that these marks have increased occupancy in meiotic cells 1.8 times (Fig. 4c), and 1.9 times in somatic cells (Fig. 4d). Since H3K27me3 modification is introduced by EZH2, we assayed the mRNA expression levels of *Ezh2* in E15.5 ovaries by using RT-qPCR. The analysis revealed that *Ezh2* expression has increased 2.2 times (Fig. 4e).

**Fig. 5:**
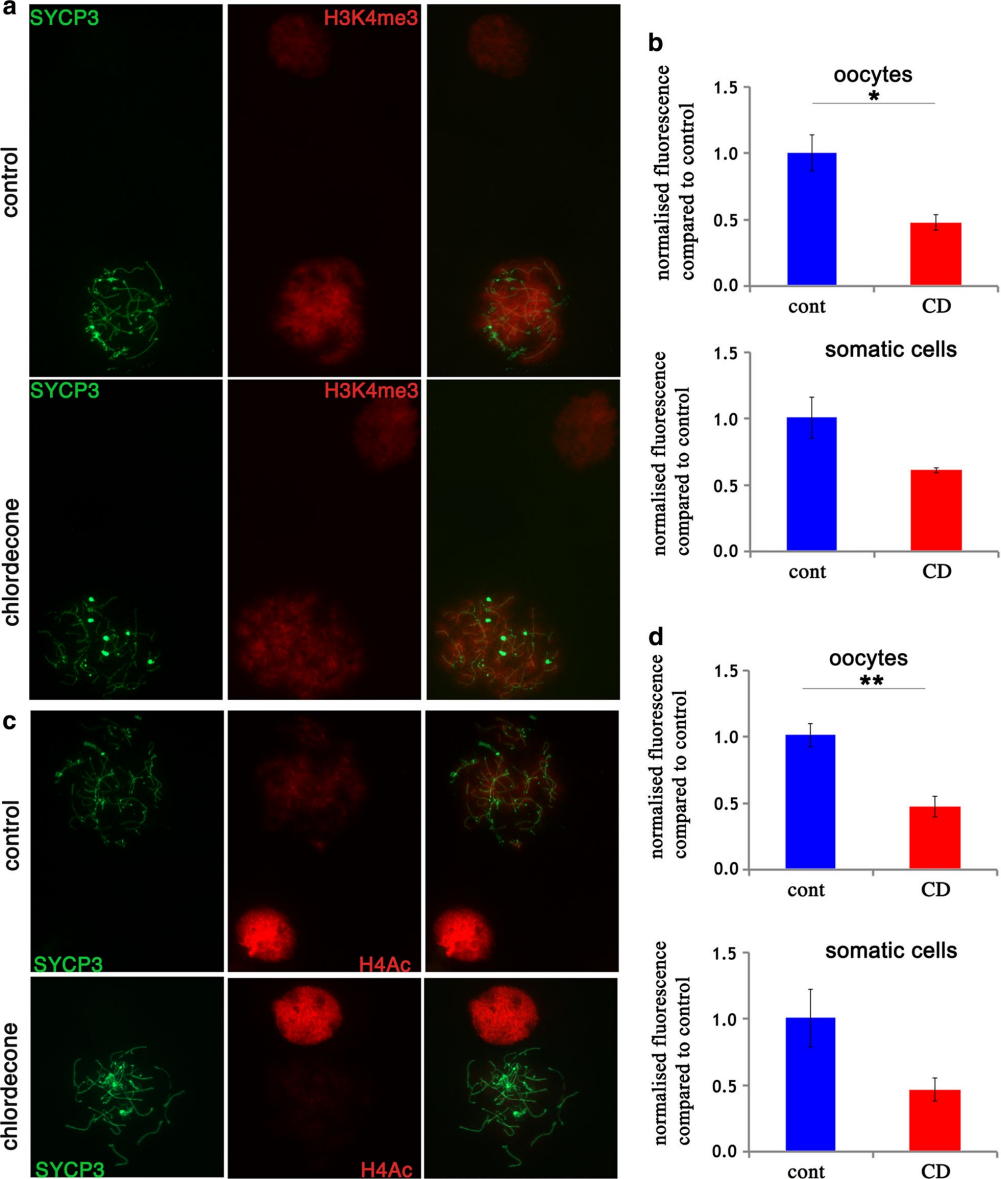
Meiosis defects are associated with altered H3K4me3 and H4Ac occupancy. **a** Surface spreads from E15.5 ovaries in *zygotene* stage and somatic cells from control (first row) and CD-exposed mice (second row) were immunostained with anti-H3K4me3 (red) and anti-SYCP3 (green) antibodies (63X magnification). **c** Surface spreads from E15.5 ovaries in *zygotene* stage and somatic cells from control and CD-exposed mice were immunostained with anti-H4 (red) and anti-SYCP3 (green) antibodies. **b**, **d** Quantitative analysis of anti-H3K4me3 and H4Ac intensities in *zygotene* stages of oocytes and in somatic cells. The analysis was performed on at least 20 oocytes from four replicates for each group, *n* = 4 for each group, *p < 0.05, **p < 0.01, ***p < 0.001, *t* test

We also analyzed the effects of CD on histone H3 trimethylation at lysine 4. Histone H3K4me3 is a pivotal mark that is associated with transcription start site, and it is also enriched in regions with open chromatin [30, 31]. We performed the analysis of H3K4me3 using immunostaining of meiotic spreads against H3K4me3 (Fig. 5a). The quantitative analysis showed that H3K4me3 intensity has decreased in oocytes 2.1 times (Fig. 5b), but not significantly affected in somatic cells (Fig. 5b).

H4 acetylation is important both for DNA repair and for chromatin-remodeling events [32]. We also analyzed histone acetylation using immunostaining of meiotic spreads against H4ac (Penta, acetylated at residues K5, K8, K12, K16) (Fig. 5c). The quantitative analysis showed that H4ac intensity has decreased 2.1 times in the CD-exposed oocytes (Fig. 5d), but no significant changes were observed in somatic cells. Our data suggest that epigenetic marks associated with transcriptional activities and chromatin remodeling were also altered upon CD exposure in embryonic oocytes.

### The effects of CD on embryonic gene expression

In our previous work that examined the effects of CD on males, we found that a large number of genes were deregulated [2]. To identify whether these genes are affected in female mice, we analyzed the expression of some of these genes in E15.5 embryonic ovaries. We found that the expression of *Rcbtb2* (regulator of chromosome condensation [33]) and *Rbpms* (RNA-binding protein critical repressor of AP-1 signaling [34]) was totally absent in E15.5-treated ovaries (Additional file 1: Fig. S3), which implies that CD targets similar genes in ovaries as well.

We also analyzed the expression of genes encoding for transcription factors important for ovarian development, FOXL2 and FOXO3. We determined that expression of *Foxo3* RNA increased 2.3-fold. In contrast, no significant changes in the expression of *Foxl2* were noted. Since CD has estrogenic effects, we tested whether exposure to CD affects the expression level of nuclear receptors such as *Esr1* and *Esr2*, but no significant alterations could be observed (Additional file 1: Fig. S3). Overall, our data demonstrate that gestational exposure to CD affects genes associated with important functions including development and chromatin remodeling.

### Exposure to CD leads to delayed puberty and affects the expression of multiple genes important for follicle development

**Fig. 6:**
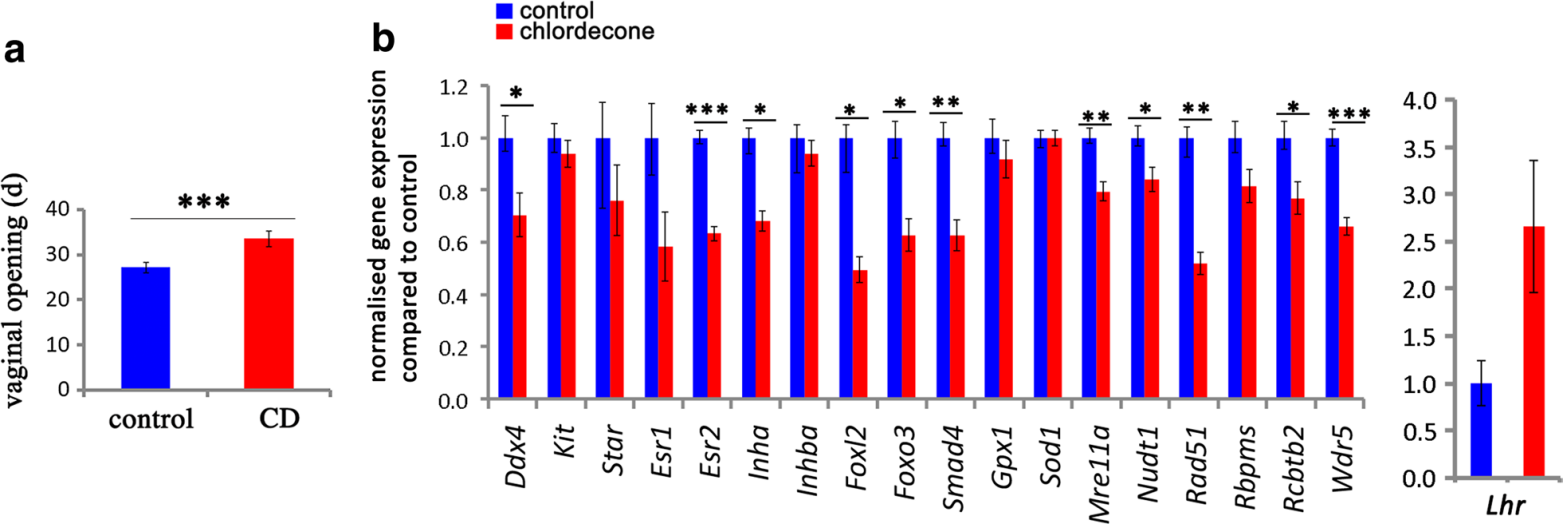
Gestational exposure to CD delays puberty and changes the gene expression. **a** Age of mice (days) at the time of vaginal opening (*n* = 15 for control, *n* = 20 for treatment, ***p < 0.001, *t* test). **b** Gene expression was analyzed by RT-qPCR using RNA from ovaries at the first estrus. The expression of genes was normalized to expression of housekeeping *Rpl37a* gene, *p < 0.05, **p < 0.01, ***p < 0.001, *t* test

To understand how embryonic exposure to CD impacts the ovarian function in adults, we analyzed the timing of vaginal opening in female rodents. We checked the opening daily in control and treated groups. We observed that there was a significant delay in the vaginal opening in CD-treated mice compared to the control group (27.1 ± 0.3 control and 33.6 ± 0.4 treatment, ***p < 0.001, *t* test) (Fig. 6a).

To explore the molecular mechanisms involved in delayed puberty, we performed RT-qPCR gene expression analysis in the ovaries dissected at first estrus. For the analysis, we selected several candidate genes, including germ cell markers (*Ddx4, Kit*), genes controlling the estrogen signaling pathway (*Star, Esr1, Esr2, Inha, Inhba)* and the genes involved in transcriptional regulation of ovarian development (*Foxl2, Foxo3, Smad4).* Because many toxic substances cause oxidative stress, we verified the expression of the genes that are involved in oxidative stress response (*Gpx1, Sod1* and *Nudt1)*. We also analyzed the expression of genes that encode for DNA repair proteins (*Mre11a, Rad51, Rcbtb2)* and the enzymes that introduce histone H3 methylation (*Wdr5*). We found a decrease in the expression of most of these genes (Fig. 6b). For example, *Esr2, Inha, Foxl2* and *Foxo3* were downregulated in the treated ovaries (Fig. 6b). Reanalysis of gene expression in the ovaries of three-month-old animals (Additional file 1: Fig. S4) showed a dramatic decrease in the expression of *Inhba* (10 times), which was associated with a strong decrease in *Esr2* (3 times) compared to the control.

Our data suggest that gestational exposure to CD leads to delayed puberty, which is associated with modified expression of genes implicated in the estrogenic signaling pathway, oxidative stress, DNA repair and histone modification.

### CD exposure affects the ovarian cell population

**Fig. 7:**
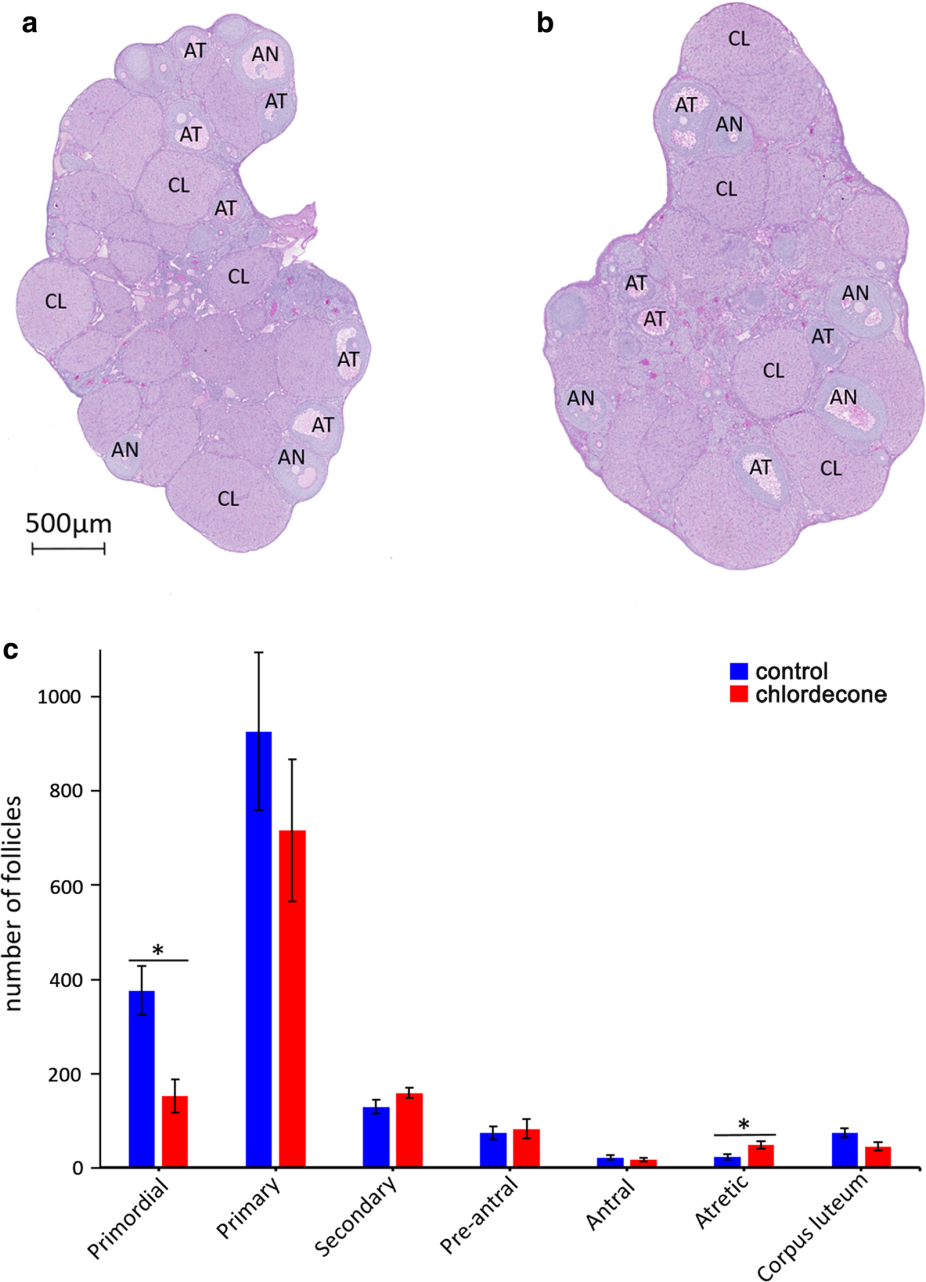
The morphological changes in ovaries promoted by CD. The representative images of ovaries in control (**a**) and treated (**b**) samples (NanoZoomer; 5X magnification). In control ovaries, most of late follicles are healthy; in contrast, in treated ovaries many atretic follicles are present. **c** The quantitative analysis of follicle numbers. The follicles were counted manually using NDP.view2 software by two independent researchers and were categorized according to the classification defined in Methods sections. We counted follicles in 4 biological replicates for control and treated group. The total numbers were compared, *n* = 4 for each group, *p < 0.05, *t* test, AT, atretic follicles, AN, normal antral follicles, CL, *corpus luteum*

To determine whether gestational exposure to CD affects the follicular population, we analyzed the histological sections of the three-month-old ovaries (Fig. 7a, b) and counted the follicle types as described in Methods section. Analysis of the follicle counts showed that there was a statistically significant decrease in the primordial follicles (926 ± 167 control, 716 ± 150 treatment, *p < 0.05, *t* test) but an increase in atretic follicles (23 ± 5 control, 49 ± 8 treatment, *p < 0.05, *t* test) (Fig. 7c) in treated group compared to control. The decrease in primordial follicles suggests that ovarian pool in treated ovaries has diminished. Since the level of anti-Mullerian hormone (AMH) is a prognostic marker of ovarian reserve [35], we analyzed the level of AMH by immunostaining ovarian sections (Additional file 1: Fig. S5A); the quantitative analysis showed a decrease in AMH in treated samples by nearly 30% (Additional file 1: Fig. S5B). In addition to this, we found that the weight of treated ovaries has decreased by nearly 25% compared to control (Additional file 1: Fig. S6).

In summary, our data demonstrate that exposure to CD leads to a decrease in primordial ovarian follicle population and an increase in atretic follicles, suggesting that CD affects the constitution of ovarian follicle pool and folliculogenesis.

### Gestational exposure to CD reduces H3K4me3 and H4ac marks in adult oocytes

**Fig. 8:**
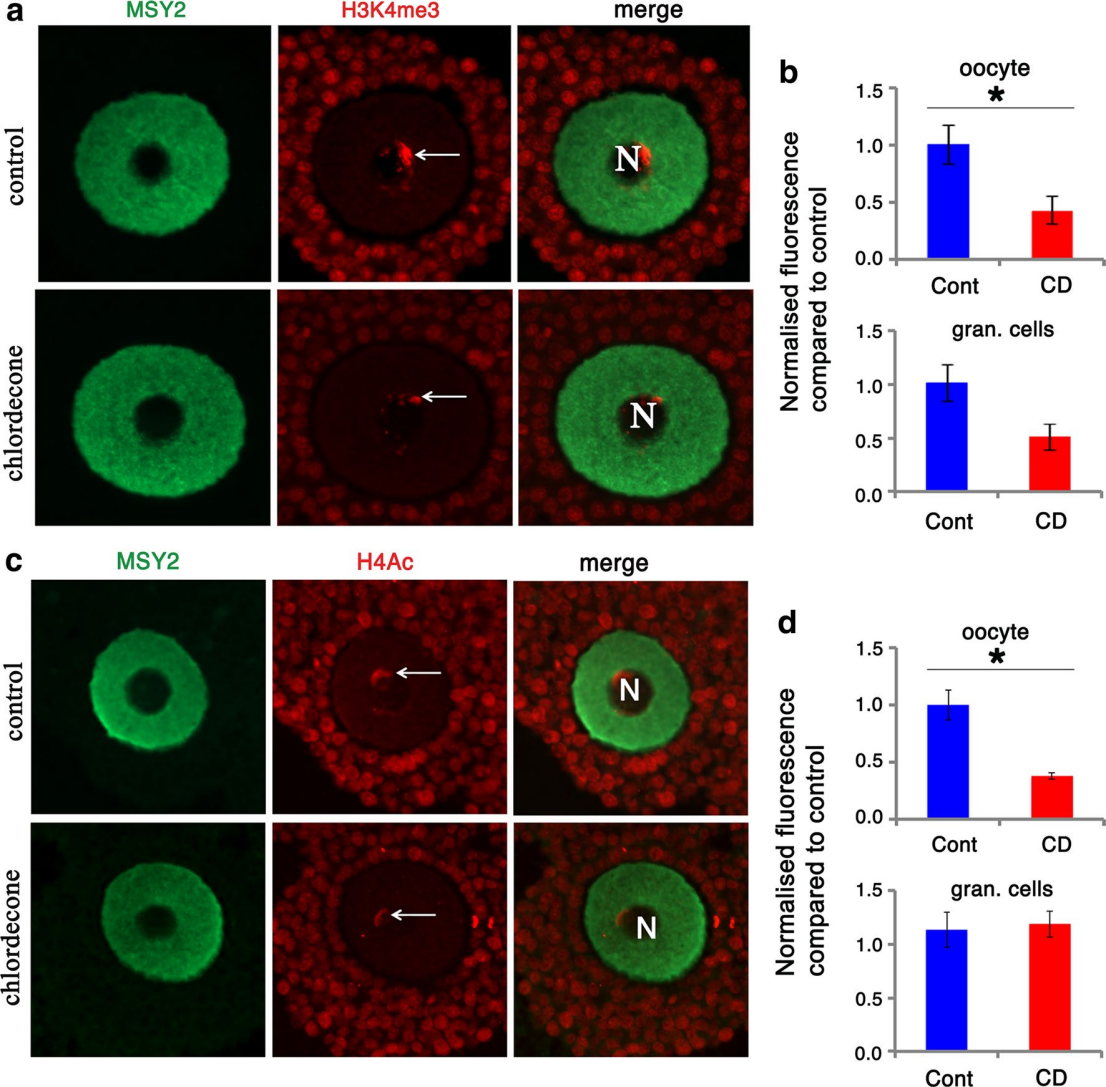
The decrease in histone H3K4me3 and H4 acetylation levels in adult ovaries following gestational CD exposure. Exposure to CD affects histone H3K4me3 and H4Ac levels in adult ovaries. **a** Representative images of control (top) or treated oocyte (bottom) immunostained by MSY2 (oocyte marker, green) or H3K4me3 (red) (40X magnification). **b** Quantitative analysis of H3K4me3 in oocytes and the surrounding granulosa cells, *n* = 4 for each group, *p < 0.05, **p < 0.01, *t* test. **c** Representative images of antral follicles immunostained for acetylated histone 4 (red). Note that in control samples homogenous nuclear staining is observed, while in treated samples clusters of bright dots are visible. **d** Quantitative analysis of H4 acetylation in granulosa cells and oocytes of control and CD-treated samples, *n* = 4 for each group, *p < 0.05, *t* test

To reveal the effects of CD on epigenetic modifications in adult mice, we performed immunostaining of paraffin sections of ovaries from three-month-old animals. We chose to analyze histone H3K4me3 marks that are normally enriched at open chromatin regions including actively transcribed genes [36, 37]. These marks appear as bright nuclear staining around the chromosomes. The marks were detected in granulosa and theca cells; they were practically absent in the oocytes of the early type of follicles, except for late antral follicles at fully grown oocytes, where the staining appeared at the periphery of the nucleus (Fig. 8a). The quantitative analysis of immunofluorescence showed a decrease in H3K4me3 marks in fully grown oocytes 2.0 times (Fig. 8b) but not in surrounding granulosa cells (Fig. 8b) of treated ovaries.

H4 acetylation is important for DNA repair [38]. To determine whether CD exposure had any impact on H4 acetylation, we immunostained ovarian sections using an antibody against hyperacetylated histone H4 (Fig. 8c), and the signal was detectable only in fully grown oocytes. We performed analysis of this mark only in fully growing oocytes and in surrounding granulosa cells. The quantitative analysis of immunofluorescence showed 2.6 times decrease in H4 acetylation in treated oocytes compared to control with no change in surrounding granulosa cells (Fig. 8d).

**Fig. 9:**
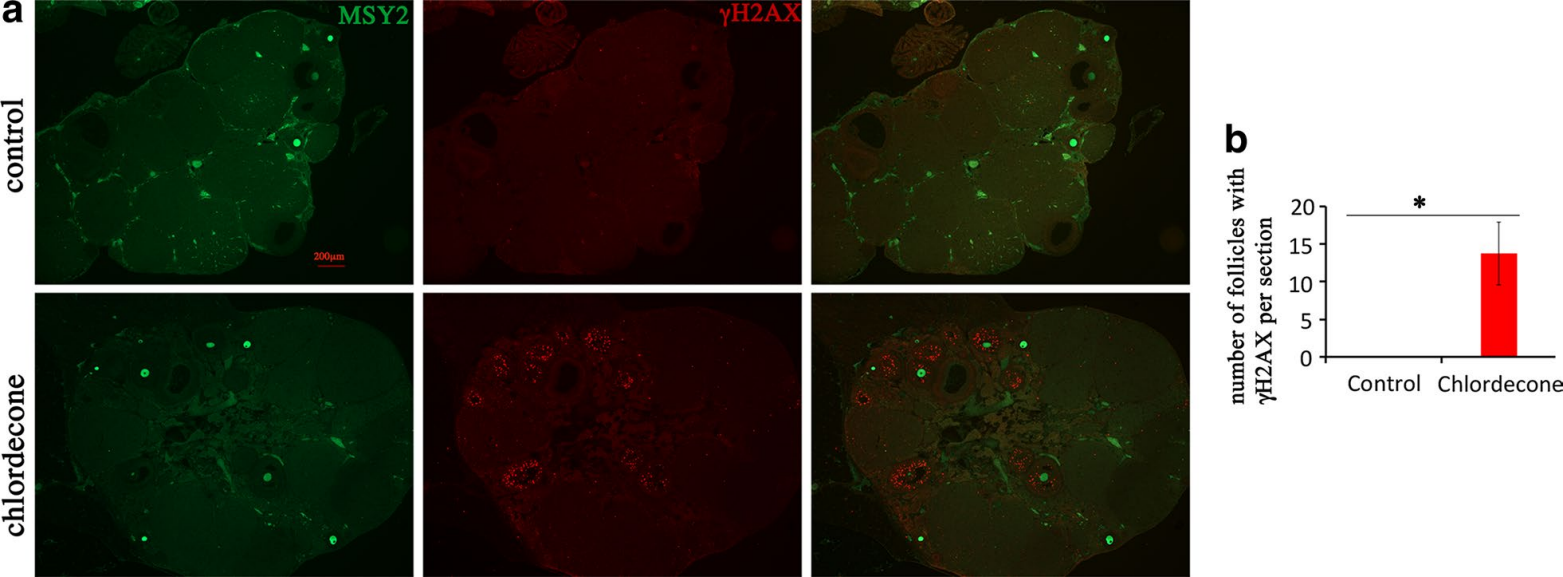
Gestational CD exposure leads to an increase in γH2AX in adult ovaries. **a** Representative images of γH2AX (red) or MSY2 (green) (5X magnification). Note that in control samples γH2AX staining is absent; in treated samples, γH2AX staining appears as dots mainly in late (preantral and antral) follicles. **b** Quantitative analyses of γH2AX staining, *n* = 4 for each group, *p < 0.05, *t* test

The non-canonical histone H2AX is phosphorylated at serine-139 position (γH2AX) when DSBs are formed; thus, the presence of γH2AX reflects the presence of DSBs. To reveal whether CD induces DSBs in adult ovaries, we coimmunostained ovarian sections using antibodies against γH2AX and MSY2 (a marker of oocytes). In treated ovaries, γH2AX staining appeared as clusters in granulosa cells of late follicles, suggesting that the follicles have some DNA damage that is not repaired. No clusters of γH2AX were detected in control samples, which indicate that observed effects are specific to CD exposure (Fig. 9a). The quantitative analysis of γH2AX-positive follicles (which have more than 5 γH2AX-positive cells) revealed that in treated oocytes, on average, 13.8 follicles per section have DNA-damaging marks (Fig. 9b).

To sum up, our analysis reveals that gestational exposure to CD associates with the decreased level of histone H3 trimethylation and H4 acetylation in fully grown oocytes.

### The genome-wide analysis of H3K4me3 revealed changes in genes important for regulating imprinting and pluripotency

**Fig. 10:**
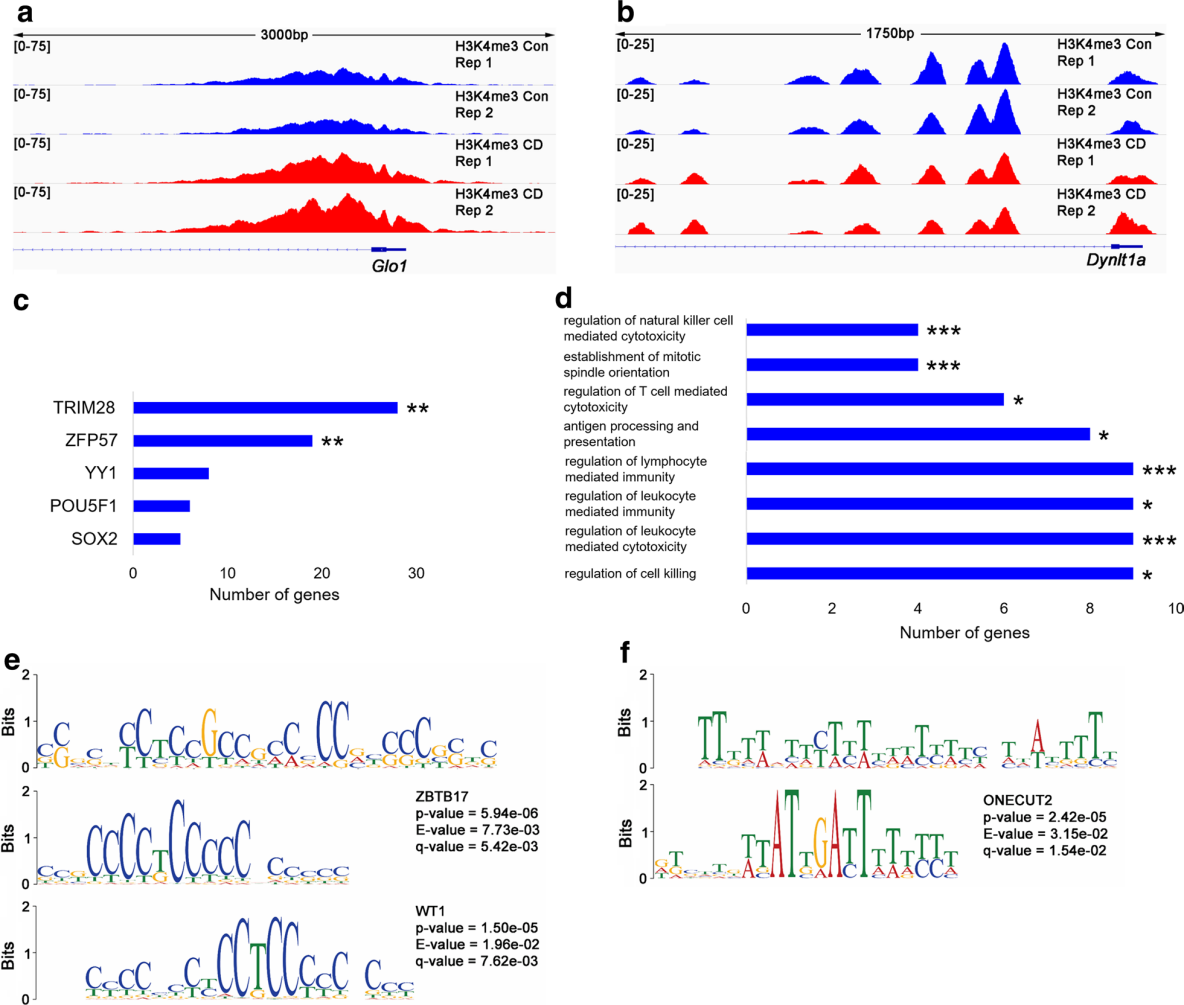
ChIP-Seq analysis of histone H3K4me3 in ovaries following CD treatment. Differential H3K4me3 peaks in the *Glo1* (**a**) and *Dynlt1a* (**b**) genes. **c** Enrichment in GO terms located within the differential peaks identified by ChEA. Terms are sorted based on adjusted *p* values indicated at the end of each bar. **d** The gene ontology (GO) terms are sorted based on *p* value, calculated by GREAT; *p* values are indicated against the GO terms in the table, Fisher’s exact test. **e** First motif identified by MEME-CHIP. WT1 and ZBTB17 binding sites are shown below the 28-mer motif. **f** Second motif identified by MEME-CHIP. ONECUT2 binding site is shown below the 29-mer motif

To reveal the regions that have altered epigenetic marks, we performed chromatin immunoprecipitation against the H3K4me3 antibody followed by high-throughput sequencing using Illumina technology (ChIP-seq, details in Methods section). The analysis of sequencing reads revealed that 151 H3K4me3 peaks were differential between control and treated groups (FC > 1.5, FDR < 0.1) (Additional file 1: Table S1). For example, H3K4me3 in *Glo1* gene that is overexpressed in many cancers [39–42] has increased (Fig. 10a), whereas in *Dynlt1a* has both decreased and increased occupancies (Fig. 10b) in treated ovary. Next, we used ChEA (ChIP Enrichment analysis) to compare our identified differential regions with published datasets [43]. The ChEA analysis revealed that targets of ZFP57 and TRIM28 were significantly enriched in differential peaks. For instance, ZFP57, a gene essential for the regulation of allele-specific expression of imprinting genes, has 19 targets (*p* value = 1.68e−05, adj. *p* value = 5.88e−03) (Fig. 10c and Additional file 1: Table S2). Among them, there were genes important for signaling (*Pik3c3*), genes playing a role in breast (*Nav3*) [44] and ovarian cancers (*Epcam*) [45]. TRIM28 has 28 targets including *Samd9* *l* and *Chd9* genes (Fig. 10c and Additional file 1: Table S3). For instance, *Dynlt1a* is a target of both ZFP57 and TRIM28 and it has differential H3K4me3 peak in its promoter (Fig. 10b).

We also assigned regions to the genes located within differential H3K4me3 peaks using GREAT and identified 185 genes that are located near the altered H3K4me3 peaks. Our analysis revealed that genes associated with mitotic spindle orientation (*Dynlt1a, Dynlt1b)* were most enriched in differential peaks (Fig. 10d).

To explore the role for transcriptional regulation in altering epigenetic modifications in the CD-exposed ovaries, we analyzed the altered H3K4me3 peaks for motif enrichment using MEME-ChIP as described in Methods section. We identified two significantly enriched motifs in altered H3K4me3 peaks; the part of the first motif is significantly similar to ZBTB17 and WT1 binding sites (*p* value = 5.94e−06 and 1.50e−05 and *q* value = 5.42e−03 and 7.62e−03, respectively) (Fig. 10e). For example, differential peak in *Cop1* gene has ZBTB17 binding site (Additional file 1: Fig. S7). In the second identified motif, part of the sequence is similar to ONECUT2 binding site (*p* value = 2.42e−05, *q* value = 1.54e−02) (Fig. 10f).

In summary, our data suggest that changes in limited regions in the mouse genome, such as genes associated with mitotic division function, could contribute to observed phenotype, such as abnormal mitosis of granulosa cells, which could lead to atresia of ovarian follicles.

## Discussion

### The embryonic effects of CD exposure

In this study, we examined the effects of CD on ovarian function. Given the potential of CD to cause transgenerational effects and the fact that humans remain exposed to CD despite discontinued use, understanding the mechanisms of CD actions on female germline is of great importance. In this study, we used a low dose of CD (100 ug/kg/day), which was previously considered to be safe [46]. This dose was used in our previous study, and we showed that it leads to transgenerational effects in males [2]. So, we asked the question whether this dose could promote changes in female reproductive system. Following exposure to CD, we analyzed the ovaries of mice at several developmental time points: at prophase I of meiosis (embryonic day 15 and 17), at first estrus (day 27 ± 1, in control) and at the age of three and five months. We found that CD causes oxidative stress in ovaries, which is manifested as increased production of 8-oxo-G in the nuclei of oocytes. It has been established that exposure to chemicals leads to the formation of excess reactive oxygen species (ROS) via several mechanisms, including the mitochondrial respiration and the detoxification pathways (reviewed in [47]). Guanine nucleotide is particularly vulnerable to oxidation and could lead to the formation of 8-oxo-derivative [47]. The increase in the expression of *Nudt1* mRNA confirms that cells have responded to increased production of 8-oxo-G so that they could be eliminated. We believe that formation of 8-oxo-G affected meiosis because it could delay meiotic DNA repair. The higher number of DMC1 foci at 15.5 that persists until E17.5 in CD-treated oocytes could be explained by the complexity of the regulation of meiotic DSB repair. It has been found that RPA regulates the loading of DMC1 on DNA [48]. RPA-mutant mice do not form DMC1 foci in spite of the presence of DMC1 protein in the cells [48], suggesting that recombinase activity of DMC1 is regulated by protein factors. Up to date, many accessory proteins were found to cooperate with DMC1 to facilitate the homologous search and strand exchange activity, and among them are HOP2, MND1, SWI5, SFR1 and MEIOB (reviewed in [49]).

On the other hand, increased DSBs that we observed in the treated samples could also be the result of inefficient DSBs repair due to decreased levels of cellular ATP that arise from elevated oxidative stress. Most DNA repair proteins are ATP-dependent. Moreover, chromosome mobility that is essential for repair of DSBs [50, 51] as well as homologous search [52] are both ATP-dependent processes. However, another reason for the DSBs increase could be related to estrogenic properties of CD; it has been shown that estrogens are capable of inducing DSBs at gene promoters [53]. The dramatic increase in H2Aub in *zygotene* stage (where DSBs started to repair) of CD-exposed ovaries suggests that some breaks are induced via DNA damage and they are persistent. We found that changes in histone H2Aub correlate with an increase in H3K27me3. This could be due to the functional link between enzymes, which introduce these two marks [28, 29]. The increase in mRNA expression of histone methyltransferase EZH2, a catalytic unit of the polycomb repressive complex 2 (PRC2) that introduces H3K27me3 modification, is in consistence with the global increase in H3K27me3 in meiotic and somatic cells.

We also found a dramatic decrease in the expression of genes such as *Rcbtb2* and *Rbpms* in embryonic ovaries. *Rcbtb2* is essential for the formation of primary cilia in mammalian cells [33]. The presence of primary cilia has been documented in granulosa cells, antral follicles and ovarian surface epithelium [54, 55]. The major function of primary cilia is to sense a variety of external stimuli; several developmentally important signaling pathways are active in primary cilia. The absence or defects in the formation of primary cilia has been observed in epithelial ovarian cancer [55] that comprises the vast majority of the human ovarian cancers [56]. Therefore, the absence of *Rcbtb2* mRNA expression in the embryonic ovaries of CD-treated mice could have deleterious effects on ovarian signaling pathways. Among the downregulated genes, *Rbpms* has a broad expression pattern in adult ovaries [57] and is an important regulator of oocyte development [58]. Studies using Zebrafish have shown that *Rbpms* mutants fail to undergo oogenesis during sexual differentiation [58]. In humans, moderate-to-negligible protein expression of RBPMS has been observed in serous papillary ovarian cancer tissues when compared to control ovaries [59]. Moreover, proliferation and invasion of ovarian cancer cells could be impeded by inhibiting microRNA-21-associated target genes that included RBPMS, RCBT1 and ZNF608 [59]. Therefore, CD-induced decreased expression of the genes essential for ovarian functions could have detrimental effects on ovarian development.

### The effects of CD on follicle population

We found several changes in the ovaries of adult mice that had undergone in utero CD exposure. The delay in puberty and perturbed gene expression suggest that CD exposure affects signaling pathways in the ovary. Specifically, we observed a dramatic reduction in the expression of *Esr2, Inhba, Inha* and *Smad4* in the ovaries of three-month-old animals that reflect an abnormal granulosa cell state. The *Inhba, Inha* and *Smad4* genes implicated in signaling (TGF-beta) pathway play an essential role for granulosa cell growth and differentiation as well as ovarian steroidogenesis [60]. *Inha* knockout mice exhibited numerous multilayered follicles, and they are in an advanced stage compared to controls [61]. Activin B is a regulator of estrogens in the mouse ovary [62]. Decreased *Esr2* expression observed in our study could be due to reduced activin B levels. On the other hand, changes in *Esr2* are associated with changes in *Foxl2* expression. It has been shown that FOXL2 is required for efficient gene regulation by steroid receptors in follicular cells, with ESR2 being the main vector of estradiol signaling [63]. The dramatic decrease in *Esr2* in three-month-old ovaries and delayed puberty suggests that CD causes changes in the female reproductive system, and it seems to occur via estrogenic signaling pathways. In summary, profound changes in gene expression suggest underlying defects in granulosa cell development and function, which could explain ovarian pathology (e.g., the presence of a large number of atretic follicles and the decrease in primordial follicles).

### Epigenetic effects in adult ovary

We found a decrease in H3K4me3 staining in fully grown oocytes. As discussed in Introduction, growing oocytes gain broad domains of H3K4me3 during maturation, and these domains are essential for the normal development of the organism. These regions are also candidates for transgenerational inheritance because some of the broad domains are preserved in embryos suggesting their importance in development. Therefore, changes in the epigenetic state could perturb the future organism. However, the exact mechanism through which this occurs is yet to be identified. We found that expression of *Wdr5* has decreased. WDR5 is a part of the MLL2 histone methyltransferase complex and it gets recruited to the promoters of ESR1 target genes upon estrogen stimulation [64]. The exact role of WDR5 in the ovary is not investigated. However, an abundance of *WDR5* transcript was observed in porcine oocytes at the germinal vesicle stage, followed by a stage-specific expression pattern in early embryos [65]. Knockdown of *WDR5* using siRNA had an impact on the expression of several pluripotent genes along with DNA repair defects in blastocysts, indicating that *WDR5* is possibly a maternal-effect gene that is crucial for early development [65–67]. Therefore, it is conceivable that decreased expression of *Wdr5* caused by a reduction in ERs contributed to the global decrease in H3K4me3 levels and could possibly have an effect on development.

We found that altered H3K4me3 marks are enriched in gene targets of ZFP57 and TRIM28 (also known as KAP1). ZFP57 and TRIM28 are both required for maintaining the repressive DNA methylation and H3-lysine-9-trimethylation (H3K9me3) at imprint control regions (ICRs) [68, 69]. TRIM28 is involved in heterochromatin formation during the process of reprogramming of mouse somatic cells into induced pluripotent stem cells, suggesting its role in reprogramming [70]. In our previous study on transgenerational inheritance in males exposed to CD, we found that altered H3K4me3 regions were also enriched in ZFP57 and TRIM28 targets and the changes were detected up to third generations [2]. It is likely that the changes were triggered during somatic-to-germline transition, and it seems to be the mechanism of CD action on female lineage as well. Importantly, haploinsufficiency of *Samd9* *l* gene, another target of ZFP57, causes myeloid malignancies in mice mimicking human diseases with monosomy 7 [71]. *Chd9* is also a target of ZFP57, and it is highly expressed in oocytes where it plays an important role in the acquisition of a highly loosened chromatin structure [72]. We found epigenetic changes in genes important for mitotic function (e.g., *Dynlt1f*), which could be implicated in abnormal granulosa cell division. It is conceivable that decreased histone H3K4me3 intensity in oocyte nuclei could be affected by chromatin structure. Further work is required to confirm this.

### How embryonic effects of CD on ovary could affect oocyte maturation?

The decrease in primordial follicle pool suggests that some of these follicles were eliminated during the late prophase I. It was proposed that checkpoint activity (CHK2) gets triggered in oocytes that carry more than 10 DSBs in late prophase I, and their repair is inhibited by HORMAD1/2 by preventing intersister recombination [73]. In addition, low levels of H3K4me3 in embryonic oocytes and in adult growing oocytes suggest that the epigenetic state changes might have got altered in utero which sustained up to adulthood. We believe that a decrease in primordial follicles could be explained by prophase I meiotic defects; these oocytes could be eliminated and leads to lower primordial follicle population. The increase in atretic follicles could be explained by the fact that gestational exposure during SGT transition introduces epigenetic changes in important genes, which are implicated in cell division. Indeed, our ChIP-seq data showed that only limited regions have altered H3K4me3 marks, but these regions contain genes important for mitotic spindle orientation, chromatin structure, regulation of pluripotency and reprogramming. We believe that these changes occurred during early development and preserved at limited regions in the adults. We identified binding sites for important developmental genes such *Wt1, Zbtb17* and *Onecut2*. It is conceivable that exposure to CD affects these transcriptional factors and these factors, in turn, could change the transcription activities of their target genes.

In summary, exposure to a low dose of CD leads to meiosis defects and decreases the oocytes stockpile. Our results demonstrate that histone epigenetic marks could be one of the responsible factors for the effects of CD on adult ovaries.

## Methods

### Animal treatment

We used the same dose of CD as reported in our previous study [2]. The dose that we used is relatively a low dose (100 µkg/kg/day) and does not cause general toxic effects in mice. The exposure window was embryonic day E6.5 to E15.5, which corresponds to somatic-to-germline transition. Briefly, CD was suspended in olive oil and administered via oral gavage at a dose of 100 μg/kg/day in a volume of 150 μl for each mouse. The control mice were treated with the same volume of oil. Ovaries were analyzed at embryonic day E15 and E17.5, and at adult stage at first estrus (day 27 ± 1, in control) and in three- and five-month-old animals.

### Preparation and immunostaining of structurally preserved nuclei

Structurally preserved nuclei (SPN) for three-dimensional analysis were prepared by cutting fresh E15.5 ovarian tissues in DMEM medium (Life Technologies, GIBCO) with 0.5% protease inhibitor. The ovary suspensions were mixed with equal amounts of 3.7% (vol/vol) paraformaldehyde and 0.1 M sucrose and spread on glass slides; the slides were air-dried and kept at −80 °C. Slides were washed several times with PBS before use and 2 min with 0.1 M glycine in PBS to remove the traces of paraformaldehyde. Slides were permeabilized during 30 min in PBS/0.5% Triton at RT, washed with PBS and blocked for 30 min in solution containing 0.1% (v/v) donkey serum, 0.03% (w/v) BSA, and 0.005% (v/v) Triton X-100 in PBS. SPN were immunostained with mouse monoclonal 8-oxo-G (1:100, Merck Millipore, MAB3560) and rabbit polyclonal SYCP3 (1:100, Santa Cruz, SC-33195) antibodies at 37C for 1 h followed by several washes and incubation with fluorescent Alexa antibodies. Z-stacks were acquired with 500 nm step; 21 individual planes were taken for each individual channel, for DAPI (Blue, 350 nm), SYCP3 (Green, 488 nm) and 8-oxo-G (Red, 594 nm) using Zen Pro (version 2.3) program. All images for control and exposed samples were taken with fixed exposure time. Deconvolution was performed using the “Fast Iterative” algorithm provided by Zen Pro. The mean intensity images were generated for each z-stack, and the resulting images were analyzed in ImageJ v1.52n. We used the lasso tool for nucleus and cytoplasm contouring, and the integrated density immunofluorescence for each nucleus was calculated. The similar area with background was used, and the background was subtracted. We analyzed 4 independent biological replicates for control and treated groups, and at least 12 cells for each replicate at zygotene stage were analyzed. The data were plotted in excel and presented as corrected total cell fluorescence (CTCF) of normalized fluorescence for nucleus or cytoplasm compared to control ± SEM, *p < 0.05, **p < 0.01, *t* test.

### Meiotic chromosome spreads

The ovaries were dissected at E15.5 and E17.5 and kept in ice-cold PBS. The ovaries were placed in a drop of 100 mM sucrose (20 µl), disrupted carefully with tweezers and pipetted until a cell suspension was formed. The cells were transferred onto a glass slide with 100 µl of 1% paraformaldehyde containing 0.1% (v/v) Triton X-100 and were kept for 2–4 h in a humidified chamber at RT and then dried. The slides were washed 4 times for 1 min each in 0.4% (v/v) Kodak Photo-Flo solution, air-dried and kept at − 80 °C until use.

### Immunolabeling of ovarian spreads

The ovarian spreads were blocked with blocking buffer (0.1% (v/v) donkey serum, 0.03% (w/v) BSA, and 0.005% (v/v) Triton X-100 in PBS) for 20 min at 37 °C in a humidified chamber. The slides were incubated with a primary antibody diluted in blocking solution for 1 h at 37 °C in a humidified chamber, washed three times with 0.4% (v/v) Kodak Photo-Flo/PBS and incubated with a secondary fluorescent Alexa antibody diluted 1:500 for 20 min at RT. The slides were washed three times with 0.4% (v/v) Kodak Photo-Flo/PBS and mounted with Vectashield mounting media containing 5 µg/ml DAPI (Vector Laboratories, Burlingame, CA). The slides were analyzed with an epifluorescence Axio Imager M1 microscope (ZEISS, France), and the pictures were taken using a 63X or 100X objective with AxioVision Rel 4.7.1 (Zeiss, France). For DMC1 foci visualization, the slides containing E15.5 and E17.5 ovaries were incubated with primary rabbit anti-DMC1 (1:100, Santa Cruz, SC-22768) and mouse anti-SYPC3 (1:100, Santa Cruz, SC-33195) antibodies. DMC1 foci were counted manually using Photoshop, and the average number of foci per cell were compared between control and treated groups. For H2Aub visualization, the slides containing E15.5 ovaries were incubated with primary mouse anti-H2Aub (1:100, Merck Millipore, 05-678) and rabbit anti-SYPC3 (1:100, Santa Cruz, SC-33195) antibodies. For H3K27me3 visualization, the slides containing E15.5 ovaries were incubated with primary mouse anti-H3K27me3 (1:100, Abcam, ab6002) and rabbit anti-SYPC3 (1:100, Santa Cruz, SC-33195) antibodies. For H3K4me3 visualization, the slides containing E15.5 ovaries were incubated with primary rabbit anti-H3K4me3 (1:100, Millipore, 07-473) and rabbit anti-SYPC3 (1:100, Santa Cruz, SC-33195) antibodies, and for H4Ac visualization, the slides containing E15.5 ovaries were incubated with primary rabbit anti-H4penta (1:100, Millipore, 06-946) and mouse anti-SYPC3 (1:100, Santa Cruz, SC-74569) antibodies. We used minimum 4 biological replicates for control and treated groups derived from 4 different treated litters. We analyzed at least 20 cells for each biological replicate. We used ImageJ for quantitative analysis of immunofluorescence of histone marks. We measured the intensity of fluorescence by contouring the nuclei with lasso tool of ImageJ. The signals for histone marks, SYCP3, DAPI and background were measured. We calculated CTCF (corrected total cell fluorescence) as multiplication of mean value of fluorescence to cell area with background subtraction. The immunofluorescence signal was normalized to SYCP3 or DAPI signal and data presented as normalized fluorescence compared to control.

### Immunohistochemistry of sections

For immunostaining, the ovaries from control and CD-treated groups were fixed in 4% (w/v) PFA solution for 16 h, dehydrated and embedded in paraffin. The sections were deparaffinized and rehydrated, and the epitopes were unmasked in 0.01 M citrate buffer, pH 6 at 80 °C for 45 min. After washing in 1X PBS-0.05% Tween (PBS-T), the sections were incubated with rabbit anti-H3K4me3 (1:500, Merck Millipore, 07-473) and mouse anti-MSY2 (1:500, Santa Cruz, SC-3938440) antibody or rabbit polyclonal hyperacetylated histone 4 (1:500, Merck Millipore, 06-956) and mouse anti-MSY2, rabbit polyclonal anti-phosphorylated Histone H2AX (1:500, γH2AX, Trevigen, 4411-PC-100) or goat anti-AMH antibody (1:500, Santa Cruz, SC-6886). The sections with primary antibodies were incubated in PBS-T overnight at 4 °C in a humidified chamber. After washing in PBS-T, the sections were incubated with appropriate fluorescent secondary antibody (1:100) for 1 h in a humidified chamber at room temperature. The sections were counterstained with 0.001% (v/v) 4,6-diamidino-2-phenylindole dihydrochloride (DAPI) and mounted using Vectashield solution. The images were taken using an AxioImager microscope equipped with an AxioCam MRc5 camera and AxioVision software version 4.8.2 (Zeiss, Le Pecq, France) with a 5X or 40X objective lens. We analyzed intensity of IF only in antral follicles. For the analysis, we chose oocytes with visibly similar size nucleus. We analyzed minimum 4 follicles per replicate and quantitated signal intensity in oocyte and surrounding granulosa cells separately using ImageJ v1.52n. We subtracted background using the regions with no cells. The mean intensity for H3K4me3 or H4Ac signals was counted, normalized to oocyte marker MSY2 and presented as normalized mean fluorescence compared to control.

For γH2AX analysis, we analyzed sections with 5X magnification. We considered the follicle as impaired if the number of γH2AX-positive cells exceeded 5. We counted on average at least 4 sections for each biological replicate and presented the data as a number of “damaged” follicles per section.

### Quantification of germ cells and follicles

For the analysis of follicles, ovaries were fixed in Bouin solution, washed in PBS and 70% ethanol, dehydrated and embedded in paraffin. Five-micrometer sections of the entire ovaries were cut and every 5th section was placed on the slide. The sections were deparaffinized, rehydrated and stained with PAS hematoxylin. The follicles were quantified using the NDPview software. The follicles were counted when the nuclei of the oocytes were visible in the follicle. The follicles were classified as primordial when the oocyte was surrounded by a single layer of flattened granulosa cells; as primary, when at least one of the granulosa cells of the single layer became cuboidal; as secondary/preantral, when the number of granulosa cell layers exceeded one; and as antral, when the antrum cavity appeared. The follicles were classified as atretic when they contained at least 2 pyknotic granulosa cells. We analyzed on average 91 sections per control and 80 sections per CD-treated samples.

### RNA extraction and quantitative PCR

Total RNA was extracted using the RNeasy plus mini kit (Qiagen) according to the manufacturer’s instructions. For embryonic ovary analysis, RNA was extracted from pool of 4 embryonic ovaries. We used 5 biological replicates for control and 6 for treatment group analysis. For RNA analysis in first estrus mice, 8 individual ovaries from 4 different litters from each group were used. For RNA analysis in three-month-old mice, 6 individual ovaries from 6 litters of each group were used. Reverse transcription was performed with 1 µg of RNA using the iScript™ cDNA Synthesis Kit (Bio-Rad). The resulting cDNA was diluted 10 times and used for quantitative PCR. The primer sequences used for qPCR are shown in Additional file 1: Table S4. QPCR was performed using the iTaq Universal SYBR Green Supermix (Bio-Rad) according to the manufacturer**’**s instructions on a CFX384 Touch Real-Time PCR Detection system (Bio-Rad). The quantification cycle (Cq) values were calculated with Bio-Rad CFX Manager 3.1. Cq values of *Hprt* and *Dazl* (embryonic gonad) or *Rpl37a (*adult ovaries) cDNA were used for normalization. The data were analyzed and are presented as mean values of Fold Change (FC) compared to control ± SEM, *p < 0.05, **, P < 0.01, *** p < 0.001, *t* test.

### Chromatin immunoprecipitation and library preparation

We used two ovaries from different animals for each biological replicate; we used two replicates for control and treated groups. Chromatin immunoprecipitation in adult tissue was performed as previously described [2], except for small modifications. Specifically, chromatin was not dialyzed after sonication but instead diluted 6 times with ChIP-dilution buffer. We performed ChIP using 1 µg of H3K4me3 (Merck Millipore, 07-473). Immunoprecipitated DNA was purified with Qiagen MinElute kit, and 10 µg of DNA was used for library construction. Library was prepared using NEBNext^®^ Ultra™ II DNA Library Prep Kit for Illumina (E7645) and submitted to Genomic platform to perform massively parallel 50-bp sequencing in single-end mode.

### Peak calling and identification of differential peaks

All analyses were performed as previously described [2]. Approximately 77.6 million tags were derived from the anti-H3K4me3 ChIP. The resulting sequences were mapped back to the mouse mm10/Ensembl genome using Bowtie 1.0.0 with a seed length of 40. H3K4me3 mark peaks were identified using MACS 2.1.1 with two biological replicate samples, including the corresponding input, using a shift-size window of 73 bp, no model, and a *p* value threshold < 1e−05. Statistical significance was calculated using a Limma test.

### Functional annotation of ChIP-seq data

Functional annotation was performed using GREAT version 3.0.0 or EnrichR with default parameters [74]. We used ChEA to functionally annotate differential peaks. ChEA is a database of genes potentially regulated by specific transcription factors, where the data were extracted manually from different ChIP-seq experiments for each transcription factor. Motif finding was performed using MEME-ChIP [75] with the default parameters. Identified motifs were compared with known motifs using TomTom [76] with the default parameters.

### Data access

All sequencing data from this study are publicly available and have been deposited in the National Center for Biotechnology Information Gene Expression Omnibus (GEO); GSE number is GSE129897.

## Additional file

**Additional file 1:** **Fig. S1.** Gestational CD exposure increases H2Aub in embryonic ovaries. **Fig. S2.** Increased levels of H3K27me3 in meiotic cells following embryonic CD exposure. **Fig. S3.** Gestational exposure to CD leads to decrease in expression of developmental genes. **Fig. S4.** Gestational exposure to CD leads to decrease in expression of genes associated with signaling, transcription regulation and DNA repair in three-month-old ovaries. **Fig. S5.** Decrease in the levels of AMH in adult ovaries after gestational CD exposure. **Fig. S6.** Gestational exposure to CD leads to decrease in body and ovarian weights in 5-month-old animals. **Fig. S7.** CD induces increased H3K4me3 occupancy at ZBTB17 binding site. **Table S1.** H3K4me3 differential peaks identified in CD exposed ovaries. **Table S2.** Targets of ZFP57 identified by ChEA. **Table S3.** Targets of TRIM28 identified by ChEA. **Table S4.** Oligonucleotides used for RT-qPCR.
