# Supplementary material for: Ovarian dysfunction following prenatal exposure to an insecticide, chlordecone, associates with altered epigenetic features
Source: Epigenetics Chromatin. 2019 May 13;12:29. doi: 10.1186/s13072-019-0276-7 (PMC6515617; doi:10.1186/s13072-019-0276-7)
Supplement: Supplementary file 1 — Additional file 1: Fig. S1. Gestational CD exposure increases H2Aub in embryonic ovaries. Fig. S2. Increased levels of H3K27me3 in meiotic cells following embryonic CD exposure. Fig. S3. Gestational exposure to CD leads to decrease in expression of developmental genes. Fig. S4. Gestational exposure to CD leads to decrease in expression of genes associated with signaling, transcription regulation and DNA repair in three-month-old ovaries. Fig. S5. Decrease in the levels of AMH in adult ovaries after gestational CD exposure. Fig. S6. Gestational exposure to CD leads to decrease in body and ovarian weights in 5-month-old animals. Fig. S7. CD induces increased H3K4me3 occupancy at ZBTB17 binding site. Table S1. H3K4me3 differential peaks identified in CD exposed ovaries. Table S2. Targets of ZFP57 identified by ChEA. Table S3. Targets of TRIM28 identified by ChEA. Table S4. Oligonucleotides used for RT-qPCR. [file 13072_2019_276_MOESM1_ESM.pdf]

Ovarian dysfunction following prenatal exposure to an insecticide, chlordecone, associates with altered epigenetic features

Louis Legoff[1*], Ouzna Dali[1,2*], Shereen Cynthia D'Cruz[1*], Antonio Suglia[1], Aurore Gely-Pernot[1], Chloé Hémery[1], Pierre-Yves Kernanec[1], Abbassia Demmouche[2], Christine Kervarrec[1], Sergei Tevosian[3], Luc Multigner[1] and Fatima Smagulova[1]#

[1]Univ Rennes, EHESP, Inserm, Irset (Institut de recherche en santé, environnement et travail) - UMR_S 1085, F-35000, Rennes, France
[2] Laboratory of biotoxicologie, Department of biology, faculty of natural sciences and life, University Djilali Liabes Sidi Bel Abbes 22000, Algeria
[3] University of Florida, Department of Physiological Sciences Box 100144, 1333 Center Drive, 32610, Gainesville, FL, USA

* Equal contribution
\# corresponding author

Additional Files

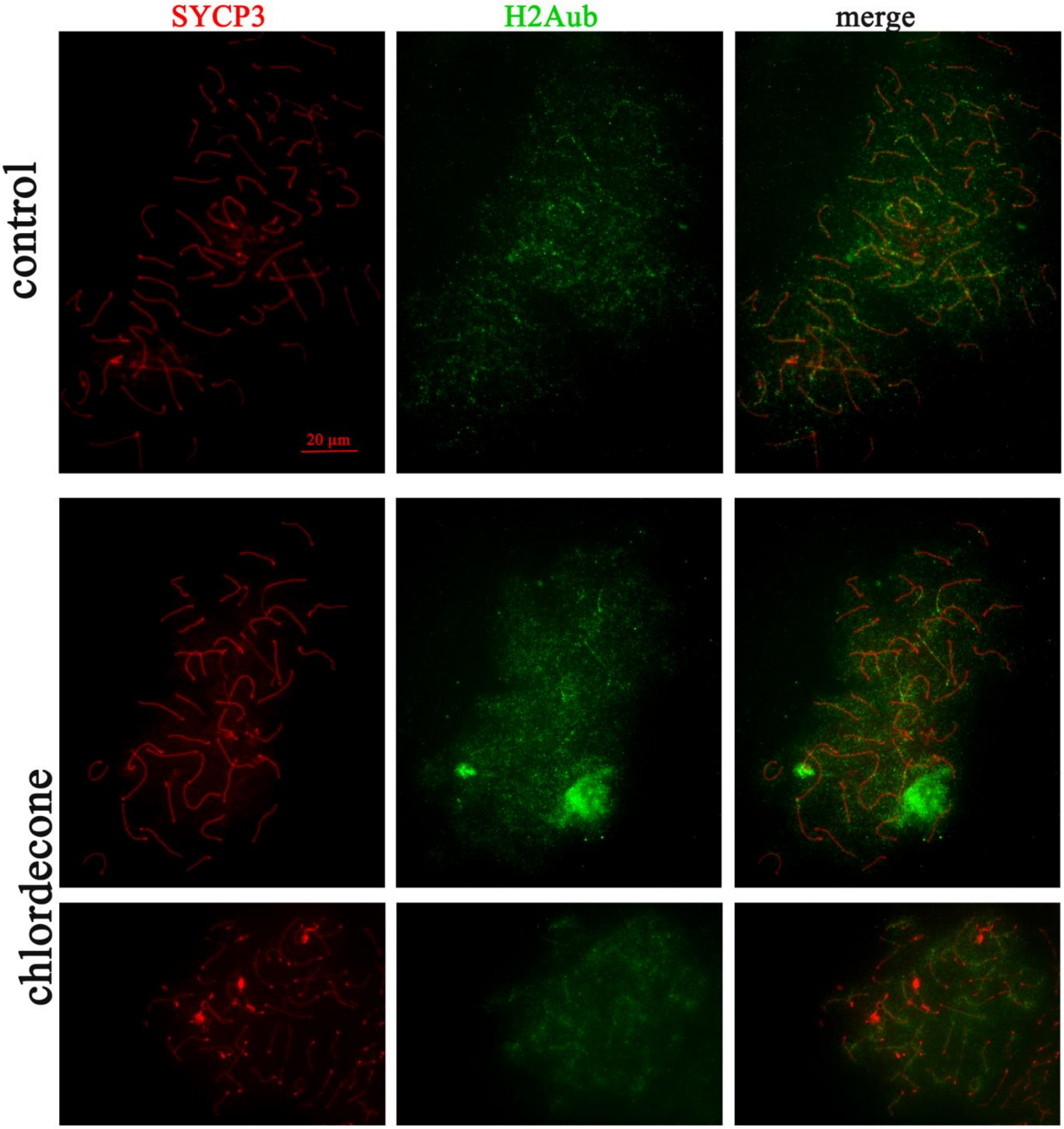

Fig. S1 Gestational CD exposure increases H2Aub in embryonic ovaries. H2Aub in surface spreads from E15.5 control (top row) and CD-exposed (bottom two row) ovaries were immunostained with anti-H2Aub (green) and anti-SYCP3 (red) antibodies.

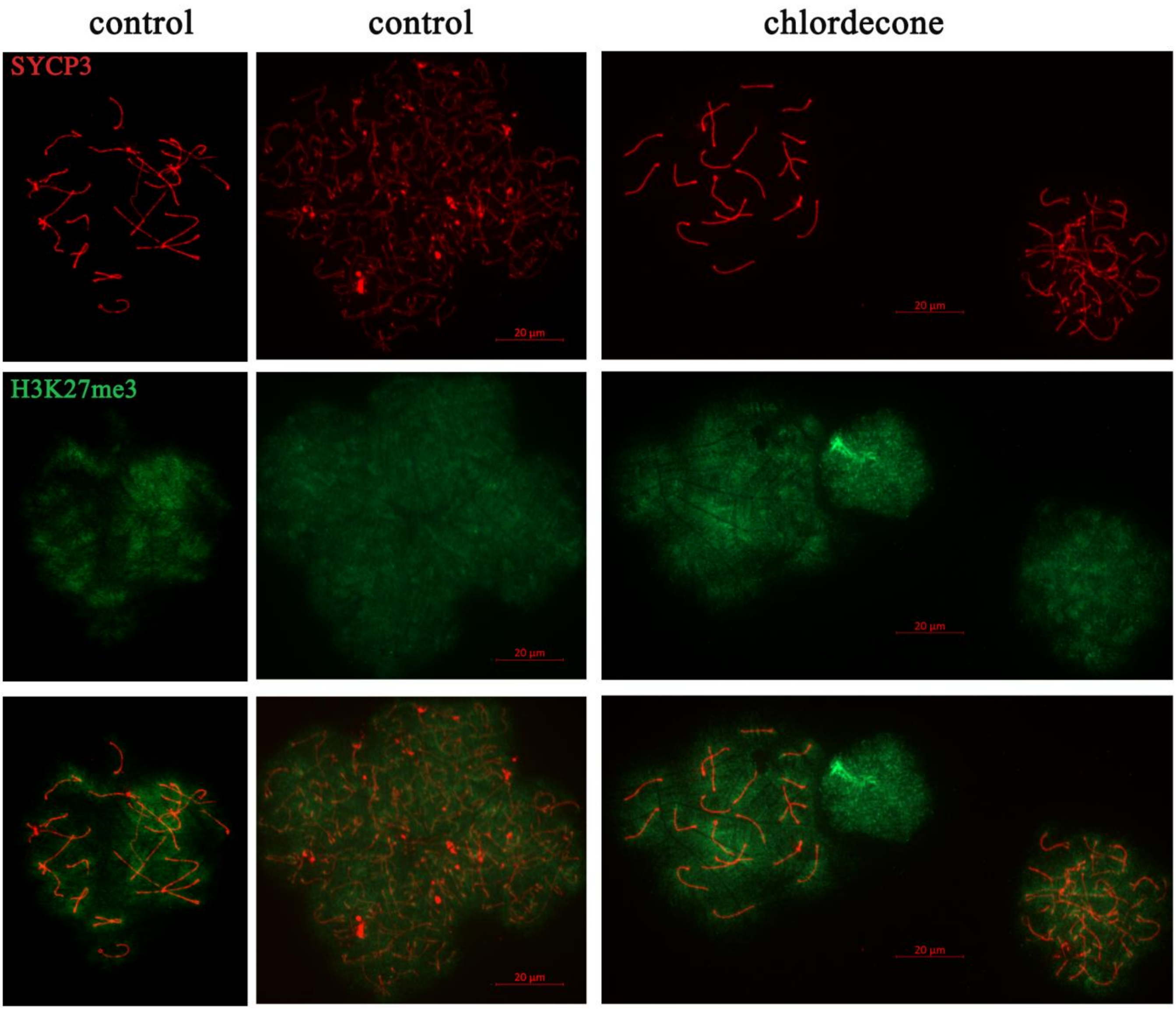

Fig. S2 Increased levels of H3K27me3 in meiotic cells following embryonic CD exposure. H3K27me3 in surface spreads from E15.5 control (two first columns) and CD-exposed (third column) ovaries were immunostained with anti-3K27me3 (green) and anti-SYCP3 (red) antibodies.

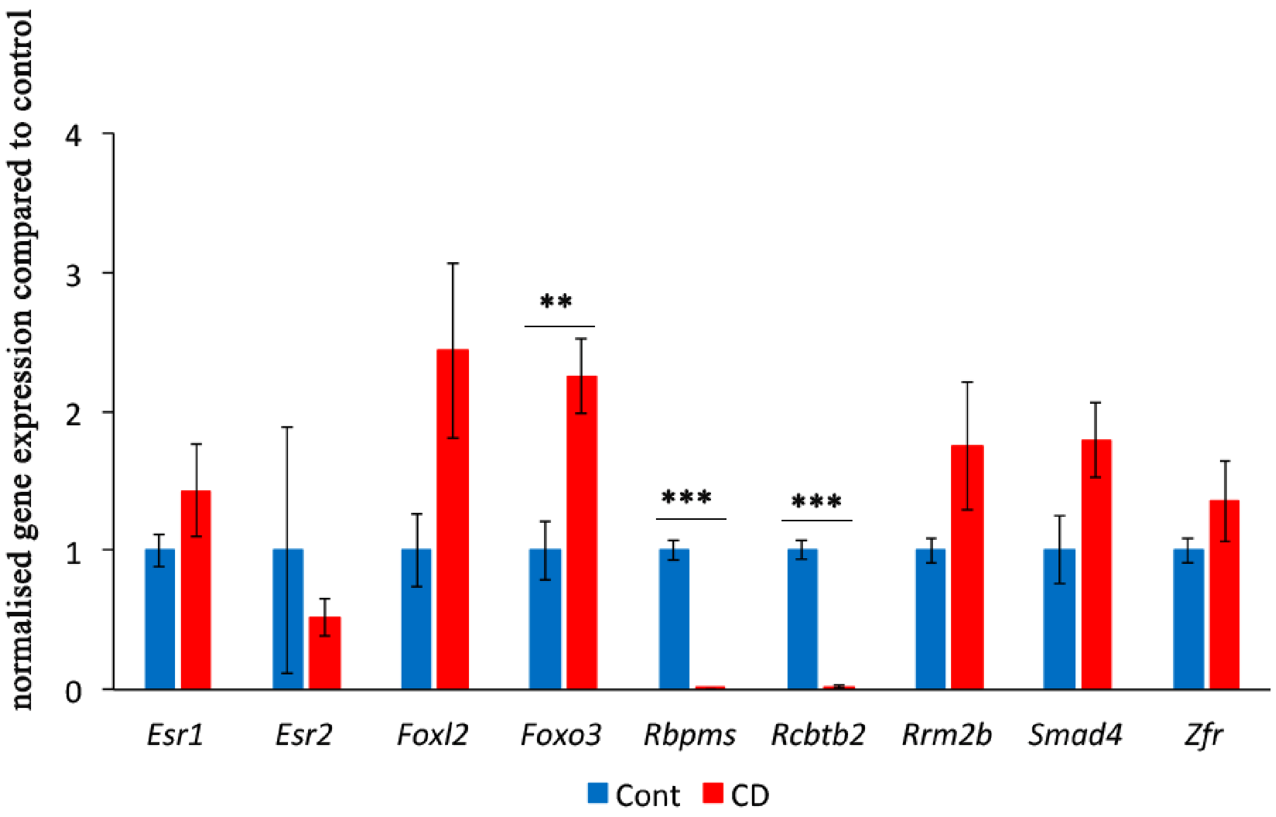

Fig. S3 Gestational exposure to CD leads to decrease in expression of developmental genes.

RT and qPCR were performed as described in Methods section using primers provided in Suppl. Table 1. The gene expressions were normalised to expression of germ cell marker*) Dazl* gene, **p<0.01, ***p<0.001, t-test.

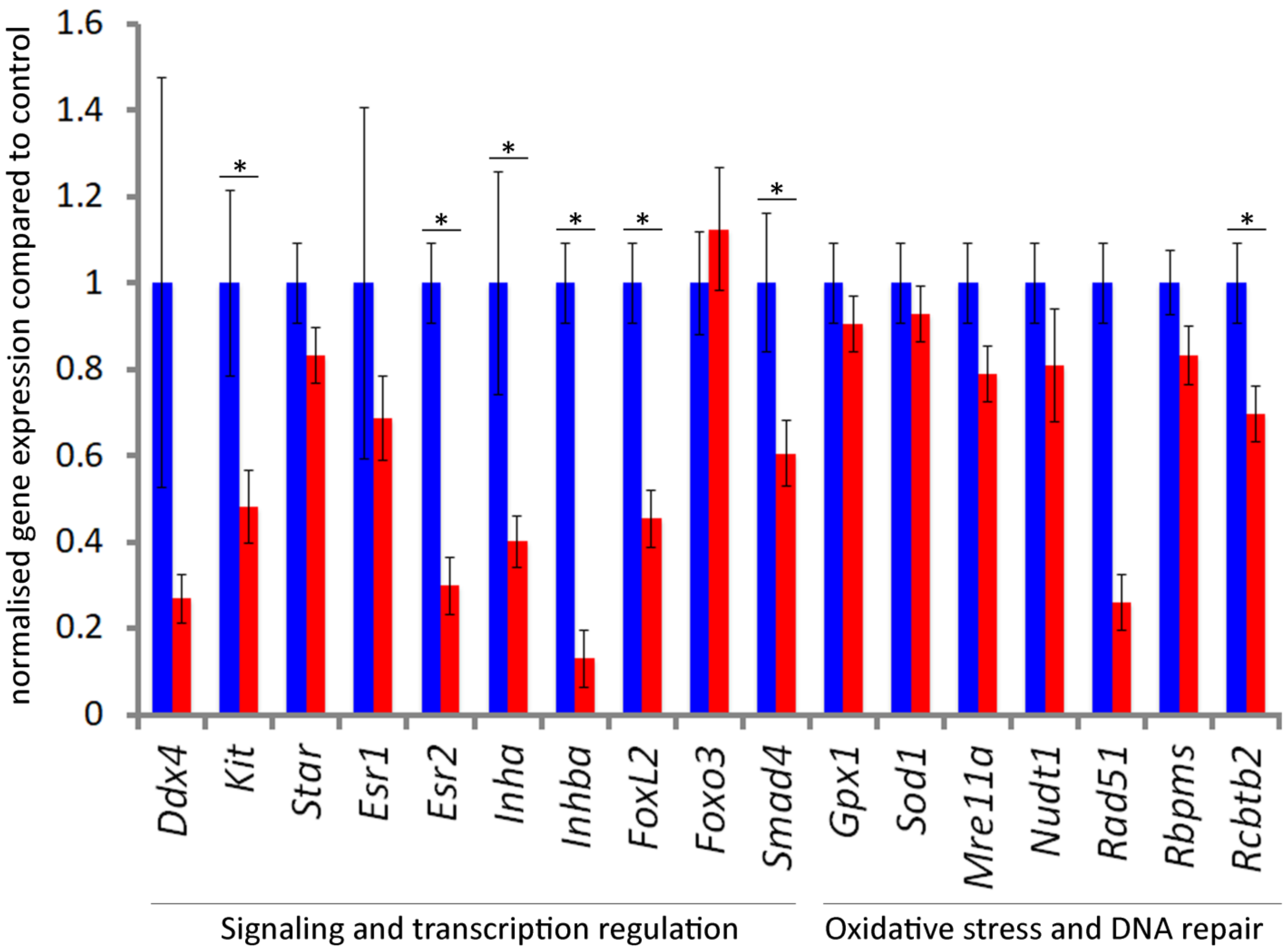

Fig. S4 Gestational exposure to CD leads to decrease in expression of genes associated with signaling, transcription regulation and DNA repair in three-month-old ovaries.

RNA was extracted from 3-month-old ovaries. RT and qPCR were performed as described in Methods sections using primers provided in Suppl. Table 1. The gene expressions were normalised to expression of housekeeping *Rpl37a* gene, *$p<0.05$, t-test.

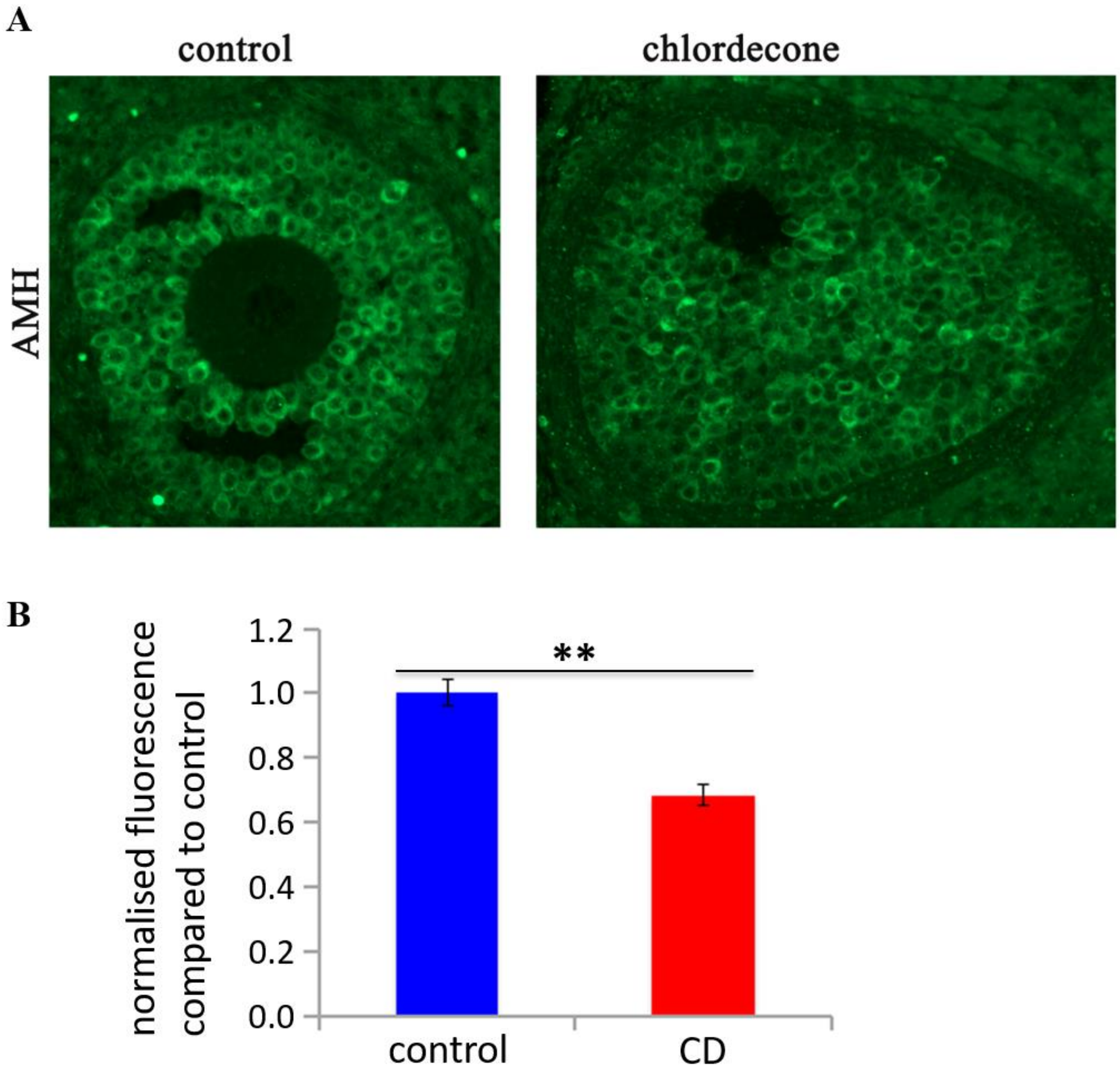

Fig. S5 Decrease in the levels of AMH in adult ovaries after gestational CD exposure.

Exposure to CD affects the AMH production in granulosa cells. (A) Representative images of control (left) or treated oocyte (right) immunostained with anti-AMH antibody (green). (B) Quantitative analysis of AMH immunostaining in granulosa cells. Sections from 4 control and 5 treatment were used for analysis. The immunofluorescence was performed as described in Method section using antibody against AMH, the images were obtained using microscope using fixed exposure time. The images were analysed using ImageJ software and the averaged value of fluorescence +/- SEM were compared, **p<0.01, t-test.

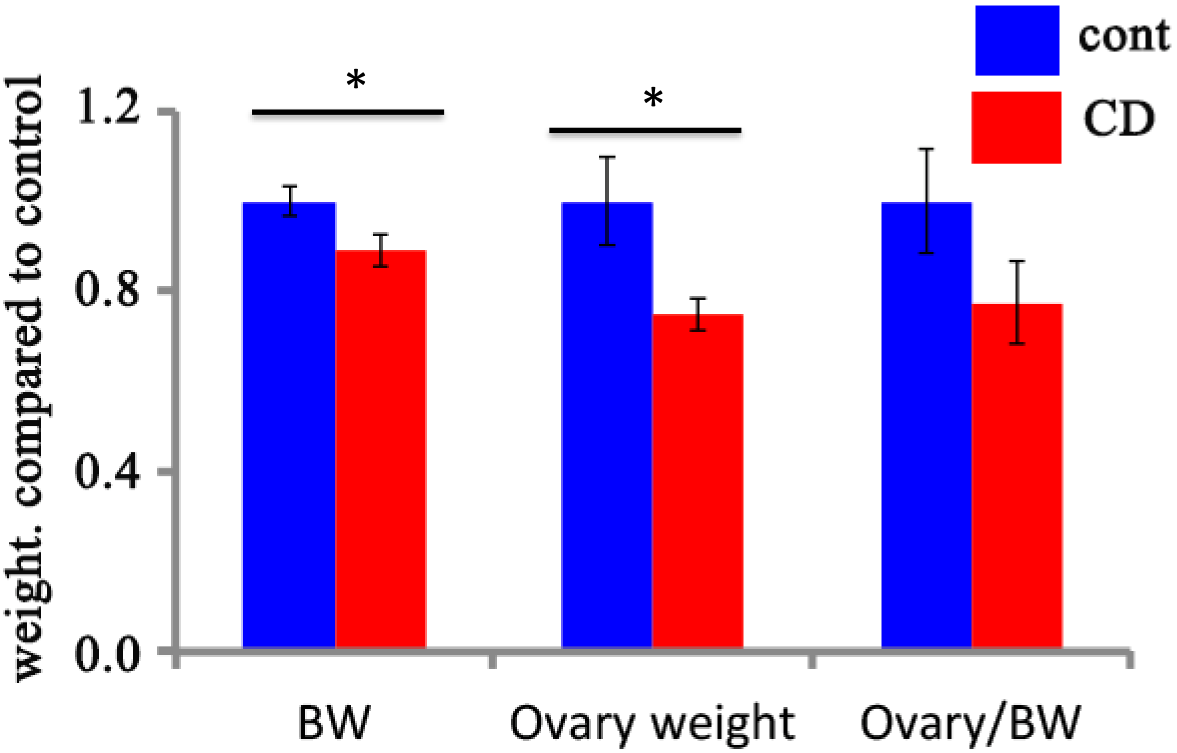

Fig. S6 Gestational exposure to CD leads to decrease in body and ovarian weights in 5-month-old animals. Ovarian sections stained by H&E from (A) control and (B) treated ovary. (C) Average body and ovary weights are presented as compared to control +/- SEM, n = 9 for control, n = 12 for treatment, *p<0.05, t-test.

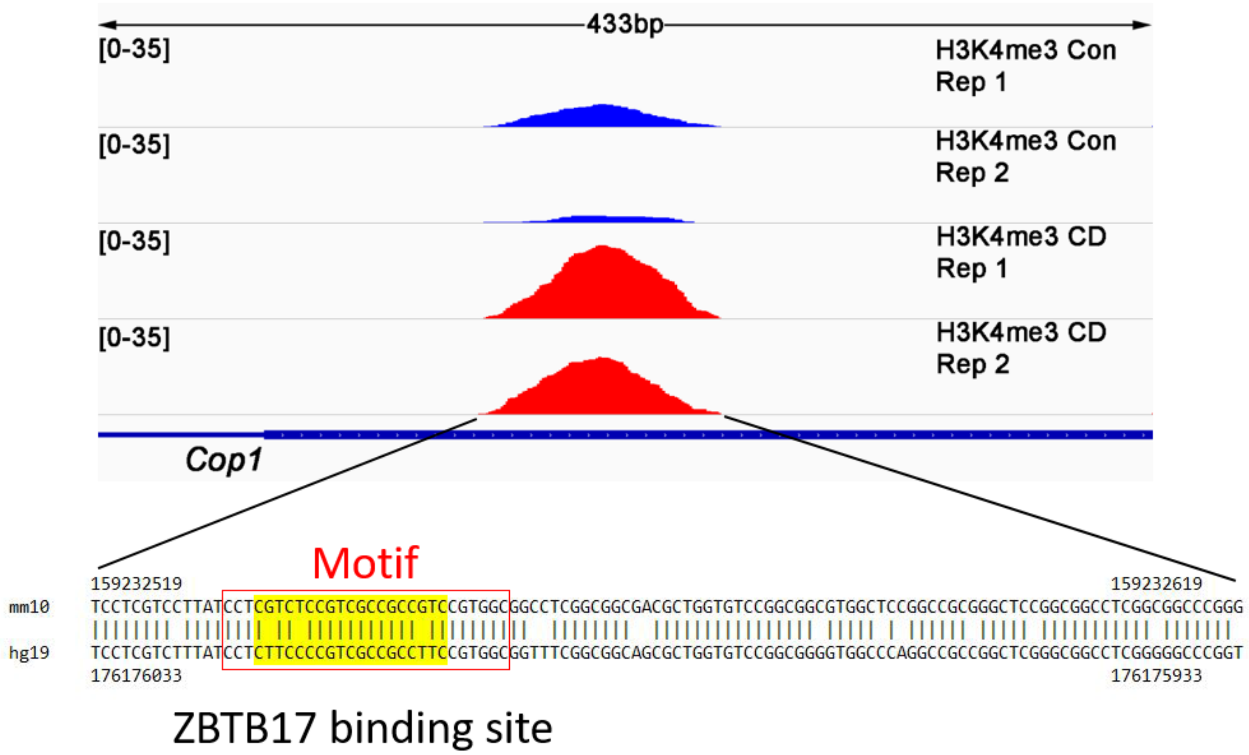

Fig. S7 CD induces increased H3K4me3 occupancy at ZBTB17 binding site Conserved ZBTB17 binding site (highlighted) was identified in H3K4me3 differential peaks in CD-exposed ovaries by MEME-CHIP.

Table S1. H3K4me3 differential peaks identified in CD exposed ovaries

Chr	Start	End	FC	Gene1	Gene2
chr1	24612703	24612792	2.81	*Gm10222*	
chr1	159232519	159232631	4.38	*Rfwd2*	
chr1	163995680	163995832	1.58	*BC055324*	*Mettl18*
chr1	170255096	170255176	1.62	*Sh2d1b1*	*Sh2d1b2*
chr1	173802146	173802281	1.58	*Ifi204*	*Mnda*
chr1	173878830	173878906	1.68	*Ifi204*	*Mnda*
chr1	173878973	173879106	1.56	*Ifi204*	*Mnda*
chr1	173879494	173879609	1.51	*Ifi204*	*Mnda*
chr1	173911324	173911421	1.65	*Ifi204*	*Mnda*
chr2	30684474	30685301	1.57	*Cstad*	*1700001O22Rik*
chr2	44923155	44923888	1.69	*Gtdc1*	*Zeb2*
chr2	89680573	89680651	1.89	*Olfr1251*	*Olfr1252*
chr2	155018509	155019055	2.38	*Gm14226*	
chr2	177252988	177253073	2.26	*Gm14410*	*Gm14409*
chr2	177956529	177956607	1.56	*Gm14326*	
chr2	180172956	180173070	1.52	*Adrm1*	*Lama5*
chr3	28765266	28765408	-3.24	*Eif5a2*	*Slc2a2*
chr3	66292404	66292849	1.55	*Veph1*	*Ptx3*
chr4	20860053	20861110	1.53	*Nkain3*	*Prdm13*
chr4	20861192	20861267	1.74	*Nkain3*	*Prdm13*
chr4	32964796	32964874	-2.40	*Ankrd6*	*Rragd*
chr4	83540426	83540529	1.76	*Ccdc171*	
chr4	117701281	117701374	2.67	*Dmap1*	*Klf17*
chr4	139330763	139331062	1.66	*Akr7a5*	*Mrto4*
chr4	145463887	145463980	1.58	*Tnfrsf8*	*Gm13225*
chr4	146604195	146604274	-2.12	*Gm13248*	*Gm13247*
chr4	147125387	147125596	-1.72	*Gm13139*	*Rex2*
chr5	25958869	25958953	1.63	*Gm5862*	*Actr3b*
chr5	26009363	26009479	1.50	*Gm5862*	*Actr3b*
chr5	26010315	26010490	1.51	*Gm5862*	*Actr3b*
chr5	101703489	101704301	2.46	*Cds1*	*Nkx6-1*
chr5	107902111	107902185	1.59	*Rpl5*	*Fam69a*
chr5	148958940	148960453	1.65	*Gm15409*	
chr6	3341102	3341346	2.54	*Samd9l*	
chr6	3396824	3397048	1.98	*Samd9l*	
chr6	119416619	119417711	1.53	*Adipor2*	
chr6	124996601	124998075	2.36	*Pianp*	
chr6	148636576	148636703	1.53	*Tmtc1*	*Ipo8*
chr6	148637730	148637822	1.66	*Tmtc1*	*Ipo8*
chr7	7209709	7209922	1.52	*Zfp772*	
chr7	7209979	7210055	2.13	*Zfp772*	
chr7	7278359	7278709	2.30	*Vmn2r29*	*Clcn4-2*
chr7	12903686	12904199	-2.21	*Rps5*	*Zscan22*
chr7	12948988	12950380	1.65	*Zfp324*	*2310014L17Rik*
chr7	48131922	48132176	1.91	*Mrgprx1*	*Mrgprb5*
chr7	48137226	48138590	2.34	*Mrgprx1*	*Mrgprb5*
chr8	21001662	21001760	2.61	*AY761185*	*Defa21*
chr8	21001830	21002001	2.21	*AY761185*	*Defa21*
chr8	83200821	83201115	1.65	*Ucp1*	*Tbc1d9*
chr8	84248821	84248905	1.78	*D8Ertd738e*	
chr8	90887157	90887272	1.75	*Gm6658*	*Chd9*

Table S1. H3K4me3 differential peaks identified in CD exposed ovaries (*continued*)

Chr	Start	End	FC	Gene1	Gene2
chr8	123426352	123428822	13.05	*Def8*	*Tubb3*
chr8	123428917	123429972	21.26	*Def8*	*Tubb3*
chr8	123430031	123430143	28.37	*Def8*	*Tubb3*
chr8	123430263	123430588	30.31	*Def8*	*Tubb3*
chr8	123430966	123431271	21.93	*Def8*	*Tubb3*
chr8	123431349	123432821	26.07	*Def8*	*Tubb3*
chr8	123432883	123433093	54.34	*Def8*	*Tubb3*
chr8	123433212	123433911	35.42	*Def8*	*Tubb3*
chr8	123433967	123434049	only exp	*Def8*	*Tubb3*
chr9	3199218	3199614	1.53	*4930433N12Rik*	
chr9	10291265	10291349	2.04	*Cntn5*	
chr9	78821635	78821758	2.47	*Cd109*	*Col12a1*
chr9	90118909	90119000	21.53	*Morf4l1*	
chr9	106684521	106684673	1.79	*Grm2*	*Tex264*
chr10	7474110	7474280	1.90	*Ulbp1*	
chr10	7492236	7492380	1.78	*Lrp11*	*Ulbp1*
chr10	8548761	8548957	2.21	*Ust*	*Sash1*
chr10	8549129	8549207	2.07	*Ust*	*Sash1*
chr10	8549523	8549672	1.95	*Ust*	*Sash1*
chr10	21188724	21189193	1.58	*Hbs1l*	*Myb*
chr10	22150037	22150137	1.88	*E030030I06Rik*	
chr10	22273152	22274656	1.56	*H60b*	
chr10	42501321	42501511	1.54	*Snx3*	
chr10	79321233	79321320	2.15	*Vmn2r82*	*Vmn2r81*
chr10	109921160	109921558	1.60	*Syt1*	*Nav3*
chr11	5708104	5708565	4.59	*Mrps24*	
chr11	6006785	6006907	2.45	*Ykt6*	*Camk2b*
chr11	6007101	6007276	2.10	*Ykt6*	*Camk2b*
chr11	6007570	6007763	2.34	*Ykt6*	*Camk2b*
chr11	82990745	82990845	1.85	*Slfn9*	*Slfn5*
chr11	83019903	83021074	1.55	*Slfn8*	
chr11	86814663	86814740	1.90	*Dhx40*	*Ypel2*
chr12	19044631	19044723	-1.57	*Gm5784*	*Gm3944*
chr12	23800431	23800600	-1.93	*Gm10330*	*9030624G23Rik*
chr12	24083613	24083695	1.97	*Gm10330*	*9030624G23Rik*
chr12	61993966	61994189	1.52	*Spanxn4*	*Lrfn5*
chr12	71342391	71342950	1.66	*Daam1*	*Dact1*
chr12	110858843	110858943	-1.60	*Zfp839*	*Cinp*
chr13	4608355	4609746	1.57	*Akr1e1*	
chr13	13438242	13438324	1.62	*Nid1*	
chr13	14021963	14022072	1.60	*Gm18856*	*Tbce*
chr13	23498190	23498808	1.54	*Hist1h4h*	*Btn2a2*
chr13	74591223	74591301	1.88	*Zfp825*	*Erap1*
chr13	99355495	99355586	2.56	*Mrps27*	*Map1b*
chr13	119597696	119597820	-3.81	*Gm21967*	*Gm7120*
chr13	119597975	119598078	-2.81	*Gm21967*	*Gm7120*
chr14	3208971	3209051	1.82	*D830030K20Rik*	*Gm10340*
chr14	5070875	5071049	1.52	*Gm8271*	*4930555G01Rik*
chr14	19585432	19585522	1.58	*Gm2244*	*Gm5458*
chr14	50061446	50062896	1.73	*Olfr725*	*Olfr726*
chr14	89898838	89898946	2.00	*Gm10110*	

Table S1. H3K4me3 differential peaks identified in CD exposed ovaries (*continued*)

Chr	Start	End	FC	Gene1	Gene2
chr14	120773347	120774644	2.13	*Ipo5*	*Rap2a*
chr15	82364741	82364826	2.00	*Ndufa6*	*Cyp2d22*
chr15	102004225	102004412	1.69	*Krt8*	
chr16	5226449	5226532	1.64	*AU021092*	
chr16	56795377	56795883	2.66	*Gpr128*	
chr16	90349454	90349544	only con	*Scaf4*	*Hunk*
chr16	97536408	97537252	1.97	*Mx2*	
chr17	3082771	3082878	1.54	*Scaf8*	
chr17	3082985	3083058	1.62	*Scaf8*	
chr17	6270514	6271091	-4.31	*Tmem181a*	
chr17	6316019	6316102	2.49	*Dynlt1a*	*Tmem181a*
chr17	6316755	6317094	-1.62	*Dynlt1a*	
chr17	6476058	6476273	1.90	*Dynlt1b*	*Gm2792*
chr17	6476353	6477115	1.63	*Dynlt1b*	*Gm2792*
chr17	6493051	6493128	-3.72	*Dynlt1b*	*Gm2792*
chr17	6601320	6601649	1.65	*Dynlt1c*	
chr17	6601815	6601940	1.99	*Dynlt1c*	
chr17	6602311	6602408	1.91	*Dynlt1c*	
chr17	6654913	6655044	2.02	*Dynlt1f*	
chr17	6655122	6655560	1.65	*Dynlt1f*	
chr17	6655700	6655789	1.55	*Dynlt1f*	
chr17	6960371	6960582	1.82	*Tagap1*	
chr17	6979477	6979595	2.64	*Rnaset2b*	
chr17	8146692	8146998	1.63	*Rnaset2a*	*Rsph3a*
chr17	8147893	8147991	1.82	*Rnaset2a*	
chr17	17064224	17064324	1.94	*Zfp960*	
chr17	22224841	22225033	2.75	*Gm4944*	
chr17	24427701	24427880	1.74	*Eci1*	*Dnase1l2*
chr17	30575058	30577498	1.98	*Btbd9*	
chr17	30611267	30613120	2.05	*Glo1*	
chr17	30635541	30636023	1.95	*Glp1r*	*Dnahc8*
chr17	30901211	30902232	2.81	*Glp1r*	
chr17	36031752	36031879	1.68	*H2-T24*	*H2-T23*
chr17	36042187	36042522	1.56	*Gm6034*	*H2-T22*
chr17	36120887	36120984	1.88	*H2-Bl*	*Gm7030*
chr17	36167485	36168446	1.74	*Gm8909*	
chr17	46461089	46461284	-2.01	*Gm5093*	*Ttbk1*
chr17	66019058	66019139	2.48	*Twsg1*	*Ankrd12*
chr17	87609391	87610082	1.64	*Calm2*	*Epcam*
chr18	30269220	30269303	1.51	*Pik3c3*	
chr18	30269425	30269501	4.77	*Pik3c3*	
chr18	43477686	43477782	802.75	*Eif3j2*	
chr19	9836719	9836841	1.53	*Stxbp3b*	*Scgb2a2*
chrX	50614492	50614582	1.55	*2610018G03Rik*	*Olfr1324*
chrX	52898621	52898713	-36.91	*Phf6*	*Ccdc160*
chrX	83875206	83875278	2.38	*Tsga8*	
chrY	4802885	4802968	10.44	*Gm20918*	*Gm8521*
chrY	10643730	10643835	2.04	*Gm20737*	*Gm20777*
chrY	90724495	90724576	1.67	*Erdr1*	*Gm21803*

Table S2. Targets of ZFP57 identified by ChEA

Coordinates	Genes	FC
chr1:173802147-173802281	*IFI204*	1.58
chr1:24612704-24612792	*COL19A1*	2.81
chr2:44923156-44923888	*GTDC1*	1.69
chr2:89680574-89680651	*OLFR1251*	1.89
chr4:20860054-20861110	*NKAIN3*	1.53
chr9:3199219-3199614	*4930433N12RIK*	1.53
chr10:109921161-109921558	*NAV3*	1.60
chr11:5708105-5708565	*MRPS24*	4.59
chr11:83019904-83021074	*SLFN8*	1.55
chr12:61993967-61994189	*LRFN5*	1.52
chr12:71342392-71342950	*DACT1*	1.66
chr13:74591224-74591301	*ERAP1*	1.88
chr14:19585433-19585522	*GM5458*	1.58
chr14:50061447-50062896	*OLFR726*	1.73
chr14:89898839-89898946	*GM5088*	2.00
chr17:6316020-6316102	*DYNLT1A*	2.49
chr17:6316756-6317094	*DYNLT1A*	-1.62
chr17:87609392-87610082	*EPCAM*	1.64
chr18:30269221-30269303	*PIK3C3*	1.51
chr18:30269426-30269501	*PIK3C3*	4.77
chr18:43477687-43477782	*DPYSL3*	only in CD

19 out of 1088 targets of ZFP57 were identified in differential peaks, p-value=1.98e-05, adj. p-value=5.88e-03, Fisher exact test.

Table S3. Targets of TRIM28 identified by ChEA

Coordinates	Genes	FC
chr1:173878831-173878906	*MNDAL*	1.6750284
chr1:173878974-173879106	*MNDAL*	1.5582267
chr1:173879495-173879609	*MNDAL*	1.5095978
chr1:24612704-24612792	*COL19A1*	2.81
chr2:89680574-89680651	*OLFR1251*	1.8945822
chr6:3396825-3397048	*SAMD9L*	1.9753817
chr8:21001663-21001760	*DEFA21*	2.6134173
chr8:21001831-21002001	*DEFA21*	2.2112592
chr8:90887158-90887272	*CHD9*	1.7538101
chr9:3199219-3199614	*4930433N12RIK*	1.5273381
chr10:42501322-42501511	*SNX3*	1.5415206
chr13:13438243-13438324	*NID1*	1.6205869
chr13:99355496-99355586	*MRPS27*	2.5560561
chr14:120773348-120774644	*IPO5*	2.1311604
chr14:19585433-19585522	*GM5458*	1.5750028
chr14:5070876-5071049	*4930555G01RIK*	1.5226169
chr14:89898839-89898946	*GM5088*	2.0005787
chr17:30575059-30577498	*BTBD9*	1.9795238
chr17:30611268-30613120	*GLO1*	2.047575
chr17:3082772-3082878	*PISD-PS2*	1.5433947
chr17:3082986-3083058	*PISD-PS2*	1.622633
chr17:30901212-30902232	*GLP1R*	2.8054997
chr17:36031753-36031879	*H2-T23*	1.681616
chr17:36042188-36042522	*H2-T9*	1.5574158
chr17:36120888-36120984	*H2-T10*	1.8759276
chr17:36167486-36168446	*GM8909*	1.738689
chr17:6270515-6271091	*TMEM181A*	-4.31375
chr17:6316020-6316102	*DYNLT1A*	2.4931009
chr17:6316756-6317094	*DYNLT1A*	-1.622523
chr17:6601321-6601649	*TMEM181C-PS*	1.6534504
chr17:6601816-6601940	*TMEM181C-PS*	1.9875913
chr17:6602312-6602408	*TMEM181C-PS*	1.9081248
chr17:6654914-6655044	*DYNLT1C*	2.0171066
chr17:6655123-6655560	*DYNLT1C*	1.6523212
chr17:6655701-6655789	*DYNLT1C*	1.5467736
chr17:8146693-8146998	*RNASET2A*	1.6264815
chr17:8147894-8147991	*RNASET2A*	1.8192032
chr18:43477687-43477782	*DPYSL3*	only in CD

28 out of 2000 targets of TRIM28 were identified in differential peaks, p-value=1.11e-05, adj. p-value=5.88e-03, Fisher exact test.

Table S4. Oligonucleotides used for RT-qPCR

Gene Name	Forward	Reverse
Dazl	CAGTATGTTCAGGCATATCCTC	ATTCATTGGGCAAAATATCAGC
Ddx4	ACAGGATGTCCCCGCATGGC	TCCCATGACTCGTCATCAACTGGA
Dmc1	GGCCCAGATGTTGTCACGACTC	TCAGTTCTCCTCTTCCCTTGCG
Esr1	CACGTTTCTGTCCAGCACCTTGAAGT	AGAGATGCTCCATGCCTTTGTTACTCA
Esr2	GTCAGGCACATCAGTAACAAGG	GTGAGCATTCAGCATCTCCA
Ezh2	CAAAGGATACAGACAGTGACAGAG	CCGAGAATTTGCTTCAGAGGAG
Foxl2	GCAAGGGAGGCGGGACAACAC	GAACGGGAACTTGGCTATGATGT
Foxo3	CAAACGGCTCACTTTGTCCC	TCATTCTGAACGCGCATGAA
Gpx1	TCTCTGAGGCACCACGATCC	TCTTGCCATTCTCCTGGTGTC
Hprt	TGCTGACCTGCTGGATTACA	TTATGTCCCCCGTTGACTGA
Inha	GGCGTCTGCCTCGAAGACAT	GTTGGGATGGCCGGAATACA
Inhba	CAGGAGGGCCGAAATGAATG	CGGATGGTGACTTTGGTCCTG
Kit	AGCGTCTTCCGGCACAACGG	GCCAATGAGCAGCGGCGTGA
Lhr	TGCCTTTGACAACCTCCTCA	TCGAAACATCTGGGAGGGTC
Mre11a	GAGCAGTAGTTGCCCAAGAAGA	TGTGAAGTCAGTATGTGGTGCTT
Nr5a1	TCGTGGTGGTAGTCGTCGTA	CTCCCTCTGGTCCTCTTGCT
Nudt1	CCGGATGACAGCTACTGGTTC	GCAGCGAGTAACTGAGGATCG
Rad51	ATTGGTTCCAATGGGTTTCA	GGCATGTAACAGCCAACGTA
Rbpms	TAGCATTTATGATAGCACCCTCAG	GTTACCTCCAAACTAAGGTCCC
Rcbtb2	CTGAAAGTGTACCAGTGCCA	GCTCATACCAGACAGATCCCT
Ring1a	CGGTTCTGAAGACTCTGGTGAC	CAGTAGTCTTCACGTACCGAGTCTG
Rnf8	GTGCCCACAGTTTCTGCTCC	CAGTCTCTTTGCTCTCCGTTCC
Rpl37a	TGGGGCCTGGACCTACAA	GCAGGGCTTCTACTGGTCTT
Rrm2b	CAGATTTCTAAAGCCCTCCTCA	AGTATGTATCCCTGATCATCCTCA
Smad4	TAATCGCGCATCAACGGAGA	CTGCTGCTGTCCTGGCTGAG
Sod1	GGACAATACACAAGGCTGTACCA	CAGTCACATTGCCCAGGTCTC
Star	TCTCTGCTTGGTTCTCAACTGG	AAACACCTTGCCCACATCTG
Stra8	CCACCTGCAACTCAGAAAATCCAG	TCCGGCCCTCCTGCTTTACAGAT
Wdr5	CTGAAGATGTCGGCTCAGGG	GCACAGAACAGACCAGGAACT
Zfr	TTCAGAGTTCAGCAGCAG	GCCATTTTGGCATCTTCCCC
